# Partially Hydrolyzed Poly(2-alkyl/aryl-2-oxazoline)s as Thermal Latent Curing Agents: Effect of Composition and Pendant Groups on Curing Behavior

**DOI:** 10.1021/acsomega.4c08659

**Published:** 2025-02-14

**Authors:** Saeed Salamatgharamaleki, Asu Ece Atespare, Taha Behroozi Kohlan, Mehmet Yildiz, Yusuf Ziya Menceloglu, Serkan Unal, Bekir Dizman

**Affiliations:** † Integrated Manufacturing Technologies Research and Application Center & Composite Technologies Center of Excellence, Sabanci University, Istanbul 34906, Turkey; ‡ Faculty of Engineering and Natural Sciences, Materials Science and Nano Engineering, Sabanci University, Istanbul 34956, Turkey

## Abstract

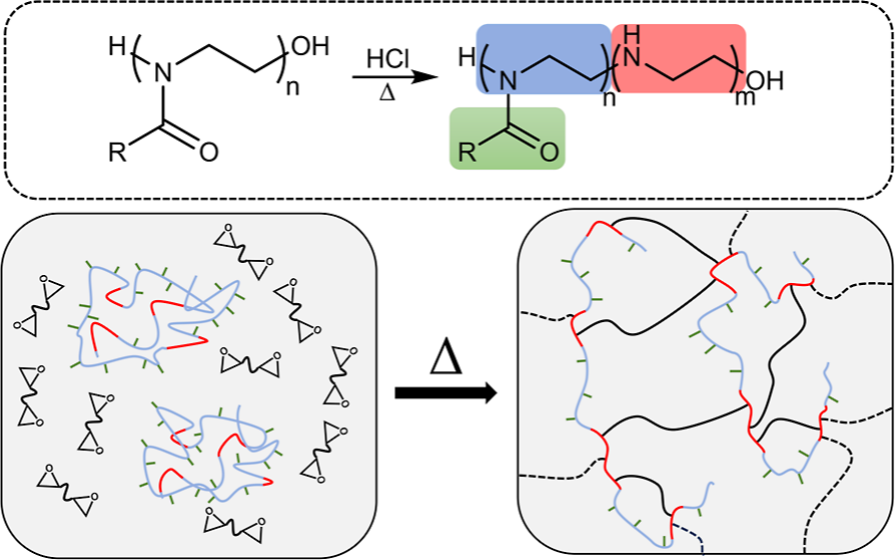

Poly(2-alkyl/aryl-2-oxazoline)–polyethylenimine (POZ–PEI) copolymers resulting from the partial hydrolysis of poly(2-alkyl/aryl-2-oxazoline)s (POZs) offer highly tunable properties. The amine groups on the PEI units are suitable for a range of postpolymerization modifications such as ring-opening of epoxides, acylation, and coupling. The reactivity of these amines can be controlled by altering the available structural variables of the copolymer. This makes these copolymers promising candidates as thermal latent curing agents (TLCs) for cross-linking of epoxides. In this paper, a range of POZ homopolymers with different alkyl/aryl pendant groups (ethyl/propyl/pentyl/phenyl) and molar masses (1000, 2000, and 5000 g/mol) were hydrolyzed at different hydrolysis ratios (25%, 50%, and 75%) to synthesize POZ–PEI copolymers. The effects of these parameters on the thermal and structural properties of the copolymers were analyzed using ^1^H NMR, FTIR, DSC, and TGA. The POZ–PEI copolymers exhibited lower glass transition temperature (*T*_g_) and decomposition temperature (*T*_d_) values in contrast to their precursor homopolymers. TLCs based on the obtained POZ–PEI copolymers were prepared and mixed with bisphenol A diglycidyl ether (DGEBA) to obtain one-component epoxy resins (OCERs). The effect of the mentioned variables on the curing behavior of the prepared OCERs was studied in terms of the enthalpy of curing, left limit temperature, and conversion. POZ–PEI-based TLCs with more hydrophobic side chains, at low hydrolysis ratios and with low molar masses, showed the best latency. PPhOZ–PEI-1 copolymer, with a *T*_g_ of 52 °C was chosen as the optimal TLC providing mainly chemical latency though steric effects and physical latency by remaining solid at room temperature. Isothermal DSC tests were performed at different temperatures to examine the stability of the resulting OCER. The results showed that this sample was stable at 40 °C for 3 h and partially cured at 60 °C. Also, the viscoelastic properties of the chosen OCER were investigated by rheology studies, namely, amplitude, frequency, and temperature sweeps. The linear viscoelastic region of the PPhOZ–PEI-1-DGEBA OCER extended up to 10% shear strain. The lowest viscosity for this OCER was observed at 104 °C, and a crossover point was seen at 118 °C. Lastly, the thermomechanical properties of the cured sample were analyzed using DMA, which showed a tan δ peak at 87.6 °C.

## 1 Introduction

Polyethylenimine (PEI) polymers have been extensively studied in literature for more than 50 years.^1^ These polymers offer excellent potential for the synthesis of functional materials by postpolymerization modifications.^1^ Depending on their synthesis pathway, PEI polymers can have structurally linear (L-PEI) and branched (b-PEI) forms, with b-PEI usually exhibiting much broader polydispersity in molar mass and structure in comparison to L-PEI.^2^ The synthesis of b-PEI was first reported in a patent in 1940s.^3^ Commercially available b-PEIs are synthesized through the cationic ring-opening polymerization of aziridine monomers catalyzed by strong acids.^4^ L-PEI and poly(2-alkyl/aryl-2-oxazoline)s (POZs) as the precursor for L-PEI were discovered simultaneously in 1966.^1^ PEIs are known for their cationically charged amines on their structure. Studies have shown that PEI polymers can react with negatively charged species and metal ions via neutralization or complexation.^5^ Owing to this characteristic, PEIs are compatible with a multitude of substrates and can be utilized as functional coatings. These polymers have been investigated as gene or drug carriers since they go through condensation when exposed to DNA or proteins and exhibit high loading capacity and transfection activity.^5^ PEI-based functional materials have been applied in therapeutic applications and other fields ranging from protein inhibition to self-healing materials.^6,7^ Partially hydrolyzed POZ–PEI copolymers have also found application in antifouling surfaces, hydrogels, and nanoparticles.^1,8^ L-PEI is mainly synthesized through complete acidic or basic hydrolysis of POZ polymers.^9^ L-PEI can also be obtained through cationic ring-opening polymerization of *N*-substituted aziridines, followed by the cleavage of side chains through hydrolysis.^1^ The bulky moieties of aziridines lower the chance of chain transfer reactions, thereby increasing the linearity of the resulting polymer. However, achieving 100% linearity through this pathway is hardly possible.^1^ Depending on the protonation state, L-PEI can exhibit different solution properties. Protonated L-PEI is soluble in water, whereas the free base form of L-PEI only shows solubility in water at temperatures above its melting point (50–68 °C).^10^ L-PEI shows pH responsiveness in an aqueous solution. Poly(2-methyl-2-oxazoline) (PMOZ) and poly(2-ethyl-2-oxazoline) (PEOZ) are generally used as precursors for L-PEI synthesis since the corresponding monomers of these polymers are commercially available, and both homopolymers are readily water-soluble.^1,10^ Acidic hydrolysis is more promising in the sense that the partially hydrolyzed species remain soluble in the aqueous solution.^1^ Since hydrolysis is conducted under an excess amount of HCl, the reaction is reported to follow pseudo-first-order kinetics.^11^ Hydrolysis under an excess amount of acid is not affected by the polymer concentration. However, the acid concentration has been shown to play a role in the kinetics of the hydrolysis reaction; hence, this variable can be manipulated to synthesize partially hydrolyzed POZs with a specific amount of ethylenimine units in a controlled way.^1^ Temperature also considerably affects the kinetics of the acidic hydrolysis of POZ polymers. With the increase in the temperature, the hydrolysis reaction also accelerates correspondingly. The hydrolysis of POZs can be stopped before full cleavage of the side chains to obtain POZ–PEI copolymers containing both oxazoline and ethylenimine units.^1^ These copolymers combine the properties of both POZ and PEI polymers, providing access to the best of the two worlds. The available secondary amine groups on the PEI units are suitable for a range of postpolymerization modifications, making these copolymers highly tunable in terms of structural, thermal, and solution properties. The partial hydrolysis of PMOZ, PEOZ, and PPrOZ has been extensively investigated and optimized in the literature.^11−14^ In addition to the abovementioned polymers, different polymers from the POZ family have been partially hydrolyzed through acidic and basic hydrolysis, namely, poly(2-phenyl-2-oxazoline) and poly(2-isoporpyl-2-oxazoline) (PiPrOZ).^15,16^ Secondary amines present in the backbone of POZ–PEIs offer high potential for modification as they function as nucleophiles. This includes modification through reactions with acid chlorides, reductive amination of aldehydes, carboxylic acid coupling via *N*,*N*′-dicyclohexylcarbodiimide (DCC)/*N*,*N*′-diisopropylcarbodiimide (DIC), urea linkage formation using isocyanate coupling, and ring opening of epoxides.^1,17−21^ The ring-opening of epoxides using secondary amines has also been used to synthesize hydrogels. In one study, Legros et al. synthesized hydrophilic hydrogels by reacting a PEOZ–PEI copolymer with 1,6-hexanediol diglycidyl ether as the cross-linker.^22^ The same group also synthesized pH-responsive degradable hydrogels and nanogels through the reaction of 1,6-hydroxyethyl disulfide-bis-diglycidyl ether with PEOZ–PEI copolymers.^20^ Nanogels synthesized in this way could be cleaved in redox-responsive behavior.

Different approaches have been employed to introduce latency in curing to amine-containing curing agents. These approaches can be divided into two categories: physical and chemical latency. In the physical latency approach, curing agents are designed to be insoluble in epoxy at room temperature due to either a high melting point or encapsulation. DICY, one of the latent curing agents with active primary amines in its structure,^23^ offers physical latency. DICY is a solid material and is insoluble in epoxy at low temperatures. DICY reaches its melting point at temperatures between 160 and 180 °C, is activated, and participates in the curing reaction. Compared to DICY, certain dihydrazides offer latency through the same mechanism while maintaining lower activation temperature ranging between 120 and 170 °C.^24−26^ However, these solid latent curing agents are incompatible with epoxy resins, resulting in processing difficulties and inhomogeneous cure.^27^ In addition, their high melting points require the usage of accelerators to lower the curing temperature, adding more complexity to manufacturing and processing steps.^27^ On the other hand, in the chemical latency approach, steric effects and reduced nucleophilicity are employed to offer latency. Chemical latency is achieved by introducing bulky moieties into the structures of curing agents to elevate the curing temperature. Kudo et al. developed a liquid latent curing agent by introducing bulky succinate moieties into the structure of imidazole.^28^ They proposed that at high temperatures, the curing agent decomposes into imidazole and fumarate via a retro-Michael addition reaction, and the imidazole starts the curing reaction.^28^ Other imidazole derivatives have been developed utilizing mechanisms such as intramolecular hydrogen bonding and flame-retardant groups to introduce latency.^29,30^ Imidazolium- and phosphonium-based ionic liquids are also used successfully as latent curing agents, which exhibit long-term stability at room temperature.^31,32^ For these latent curing agents, after the first decomposition step, the resulting anion and/or cation, depending on the ionic liquid type, initiates the epoxy resin homopolymerization.^31^

Recently, functional polymers and their modifiable properties have been employed as latent curing agents. For instance, intermolecular hydrogen bonds of polybenzoxazines can block the activity of phenolic hydroxyl groups and induce latency in epoxy mixtures up to 150 °C.^33^ Another example is the microencapsulation of imidazole thorough complexation with POZ homopolymers and copolymers to increase the pot-life of it.^34,35^ Considering the potential, POZ–PEI copolymers with variable structures and properties can also offer latency depending on the reactivity of the secondary amines present in their structure. This reactivity can be manipulated based on the different adjustable structural variables of these copolymers. Thus, we aimed to address the need for relatively easy synthesis, modifiable chemical structure, and application-targeted curing performance using POZ–PEI copolymers as latent curing agents.

In this study, POZ homopolymers were prepared by polymerization of 2-ethyl-2-oxazoline (EOZ), 2-propyl-2-oxazoline (PrOZ), 2-pentyl-2-oxazoline (PeOZ), and 2-phenyl-2-oxazoline (PhOZ) monomers.^17^ Polymerization was performed to obtain homopolymers with 1000, 2000, and 5000 g/mol molar mass. The homopolymers were then hydrolyzed under acidic conditions to obtain POZ–PEI copolymers with three different compositions targeting 25%, 50%, and 75% hydrolysis ratios. The chemical structure, molar mass, and polydispersity (*D̵*) values of the synthesized homopolymers were investigated by using ^1^H NMR, FTIR, and size exclusion chromatography (SEC) analyses. The compositions of the prepared POZ–PEI copolymers were studied by using ^1^H NMR and FTIR spectroscopy. The thermal properties of the homopolymers and copolymers in terms of crystallinity, *T*_g_, *T*_m_, and degradation temperature were studied and compared with dynamic DSC and TGA to explore the influence of pendant-group type, molar mass, and composition on these properties. In the second part, POZ–PEIs were studied as thermal latent curing agents (TLCs) for the curing of the bisphenol A diglycidyl ether (DGEBA) resin. The curing behavior of the prepared one-component epoxy resins (OCERs) containing POZ–PEI copolymers as TLCs was studied by using dynamic DSC in terms of the left limit temperature of curing, curing enthalpy, and conversion. The effects of molar mass, composition, and pendant group type of the copolymers on the curing behavior of the OCERs were examined. The best sample, in terms of left limit temperature, curing enthalpy, and ease of synthesis, was chosen for isothermal DSC tests, and the stability of this sample was studied at different temperatures.

## 2 Experimental Section

### 2.1 Materials

EOZ, PhOZ, chlorobenzene, methanol, ethanol, 37% (w/w) HCl (aq.), hexanenitrile, and hexanoic acid were purchased from Sigma-Aldrich. The monomers and chlorobenzene were dried over calcium hydride (CaH_2_) and distilled before polymerization. Butyronitrile and 2-chloroethylamine hydrochloride were purchased from ABCR. Trifluoromethanesulfonic acid (TfOH) and oxalyl chloride were purchased from Across Organics. Sodium hydroxide (NaOH) and potassium hydroxide (KOH) were purchased from ISOLAB. Ethanolamine, CaH_2_, dichloromethane (DCM), zinc acetate dihydrate (Zn(OAc)_2_·2H_2_O), triethylamine (TEA), and sodium sulfate (Na_2_SO_4_) were purchased from Merck. PrOZ and PeOZ monomers were synthesized following the procedures previously reported by our group.^36^ Unless otherwise stated, no further purification of the chemicals was performed. Deionized water was acquired from a Merck Direct-Q-3 UV.

### 2.2 Instruments

CD_3_OD and CDCl_3_ were used to record the ^1^H NMR spectra on a 500 MHz Varian spectrometer. FTIR spectra were obtained on a Thermo Scientific Nicolet iS50 FTIR spectrometer by using an attenuated total reflectance (ATR) accessory. Transmission mode was used with a resolution of 16 cm^–1^. SEC measurements were performed on a Malvern VISCOTEK GPCmax-Viscotek TDA305 instrument with a D5000-D3000-D1000-DGuard column and a refractive index detector. The temperature of the column was 55 °C, and the injection volume was 100 μL. DMF was used as an eluent at a flow rate of 0.7 mL/min, and the molar mass was calculated using both poly(methyl methacrylate) and PEOZ standards. The PEOZ standards were prepared by calibrating the instrument with five PEOZ polymers with *M*_p_ values of 500, 1000, 2000, 5000, and 10,000 Da obtained from MALDI-TOF. DSC measurements were performed using a Mettler Toledo DSC 3+ instrument with sample sizes of 8–10 mg. DSC thermograms were obtained from the second heating run at 10 °C/min from −50 to 250 °C, after the first run of heating up to 250 °C and cooling down to −50 °C at 10 °C/min, under a nitrogen atmosphere, to measure the glass transition temperatures (*T*_g_). TGA measurements were performed on a Mettler Toledo TGA/DSC 3+ instrument in the temperature range of 25–800 °C. Samples of 10–100 mg were heated at 10 °C/min under a nitrogen atmosphere at a flow rate of 100 mL/min. The samples were subjected to conditioning at 110 °C for 30 min prior to analysis. Curing studies were performed on a Mettler Toledo HP DSC 2+ instrument. The samples were weighed in the amounts of 13–20 mg on the DSC pans. The dynamic DSC thermograms were obtained from the first heating run in the range of 25–200 °C with a heating rate of 10 °C/min under a nitrogen atmosphere. Rheological analyses were performed with an Anton-Paar MCR 702 TwinDrive rheometer using a gap of 0.5 mm under static air. The amplitude sweep was conducted between 0.01% and 100% shear strain at 65 °C with a frequency of 1 Hz. The frequency sweep was performed from 0.1 to 100 Hz at 65 °C with a shear strain of 1%. The temperature sweep was conducted at 1% shear strain and a frequency of 1 Hz from 65 to 200 °C. Dynamic mechanical analysis (DMA) was carried out to investigate the thermomechanical properties of a cured sample with a NETZSCH DMA 242C instrument equipped with the three-point bending holder. The measuring frequency was 1 Hz and the oscillation amplitude was 15 μm. The temperature range was between 25 and 200 °C and the heating rate was 3 °C/min.

### 2.3 Homopolymer Synthesis Procedure

The homopolymers were synthesized and purified in accordance with our group’s previous report.^19^ Briefly, monomers and chlorobenzene were introduced into dried round-bottom flasks under a N_2_ flow. The monomer concentration was set to 4 M. TfOH was used as initiator for the polymerization. The monomer-to-initiator ratio ([M]/[I]) was fixed at 10, 20, and 50 to reach molar masses of 1000, 2000, and 5000 g/mol. The reaction temperatures and polymerization times for homopolymers are listed in Table S1. The polymerizations were terminated with methanolic solution of KOH. PEOZ, PPrOZ, PPeOZ, and PPhOZ homopolymers were obtained after workup, details of which are provided in our previous report.^22^ SEC results and reaction yields for the homopolymers are listed in Table S2.

### 2.4 Homopolymer Hydrolysis Procedure

PEOZ and PPrOZ homopolymers were weighed into a two-necked flask. 10.07 mL of deionized water and 4.93 mL of 37% (w/w) HCl (aq.) per gram of polymer was added to the flask to obtain a 4 M HCl mixture. The mixture was heated to boiling temperature (100 °C) and was held under reflux. The reaction was performed at three different time durations based on preliminary kinetic studies targeting three hydrolysis rates between 25% and 75%. After a specified time, the reaction mixture was quenched in an ice bath. Diethyl ether washing was performed to remove the resulting propionic and butyric acid from the resulting polymer. The aqueous phase was then separated and neutralized with 5 N NaOH to a pH > 10. After evaporating the water under reduced pressure, the remaining solids were dissolved in 50 mL of ethanol per gram of the starting polymer and filtered as a preliminary step to reduce the amount of salt in the mixture. The ethanol was removed under reduced pressure. 100 mL of acetonitrile per gram of homopolymer was added to the remaining solid and kept at 50 °C. The mixture was then filtered using a Büchner funnel, and the organic phase was collected. The solvent was evaporated under reduced pressure to isolate the copolymer.

**Figure 1:**
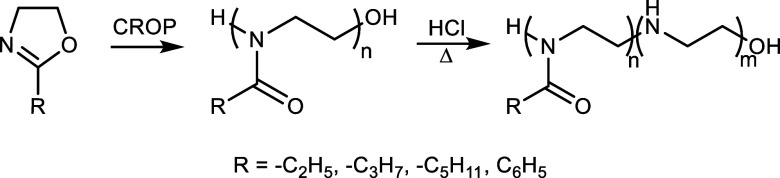
Synthesis of POZ–PEI copolymers.

To hydrolyze the PPeOZ and PPhOZ homopolymers, polymer was weighed into a two-necked flask with 16 mL of ethanol and 6.2 mL of 37% (w/w) HCl (aq.) per gram of polymer to obtain an 80:20 (v/v) ethanol: water solution with an HCl concentration of 3.37 M. The timer was started after reaching a temperature of 85 °C. The reaction was performed at three different time intervals to obtain three different hydrolysis ratios between 25% and 75%. After a specified time, the reaction mixture was quenched in an ice bath. Then, the aqueous phase was neutralized with 5 N NaOH to a pH > 10. The solvents were evaporated, and 50 mL of deionized water per gram of the starting homopolymer was added to the remaining solids. The mixture was sonicated for 10 min to dissolve the produced sodium hexanoate, sodium benzoate, and NaCl. The precipitated solids (copolymers) were then filtered and dried in an oven. Figure 1 shows the schematics of the POZ–PEI copolymer synthesis starting from the 2-oxazoline monomer.

The copolymers were coded based on their structure, molar mass of the respective starting homopolymers, and hydrolysis ratios, for example, PEOZ–PEI 1K-1. The numbering of samples as 1, 2, and 3 indicates the hydrolysis ratios of 25%, 50%, and 75% for the copolymers, respectively. The reaction time for each sample is given in Table S3.

### 2.5 Preparation of One-Component Epoxy Resins

#### 2.5.1 DSC Sample Preparation

POZ–PEI copolymers were mixed with DGEBA at an amine-to-epoxy molar ratio of 1:1 in 1.5 mL bottles to prepare POZ–PEI-based OCERs. The OCER samples were prepared to have a weight range of 200–250 mg. Solid POZ–PEI copolymers were ground before being mixed with DGEBA, while waxy copolymers were mixed as they were. The mixing was performed with a spatula for 5 min. Subsequently, 13–20 mg of the OCERs were transferred into DSC pans.

#### 2.5.2 DMA Sample Preparation

PPhOZ–PEI 1K-1 TLC was mixed with DGEBA with a molar ratio of 1:1 at 65 °C in an oil bath. The mixing was conducted by using a homogenizer at 1000 rpm for 10 min. After that, the mixture was degassed under vacuum for 15 min and casted into a preheated Teflon mold at 80 °C. The mold was put into an oven at 100 °C for 2 h to ensure that no air was present in the mixture, and curing was performed at 140 °C for 2 h. To ensure that the sample was fully cured, the sample was held at 160 °C for 2 h. Next, the sample was polished and cut into a dimension of 40 mm × 12 mm × 2.57 mm.

## 3 Results and Discussion

### 3.1 Structural Analysis of Synthesized Copolymers

**Figure 2:**
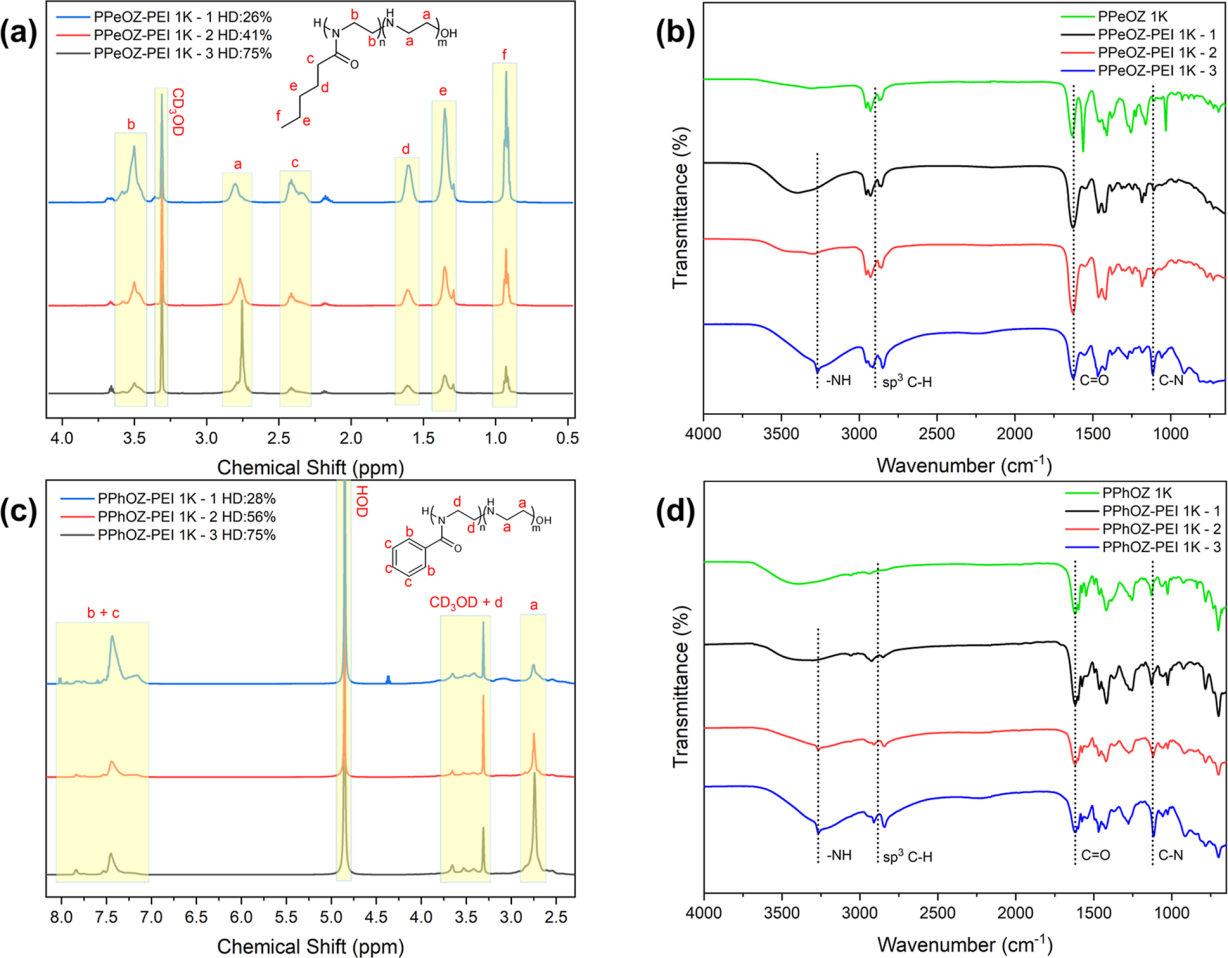
^1^H NMR spectra of PPeOZ–PEI 1K (a) and PPhOZ–PEI 1K (c) sample at different hydrolysis degrees. FTIR spectra of PPeOZ–PEI 1K (b) and PPhOZ–PEI 1K (d) at different compositions along with PPeOZ 1K and PPhOZ 1K homopolymers.

POZ homopolymers were hydrolyzed to obtain POZ–PEI copolymers with hydrolysis ratios of 25%, 50%, and 75%. The structural analysis of the copolymers was performed by ^1^H NMR and FTIR. Figure 2 shows the ^1^H NMR and FTIR spectra of PPeOZ–PEI 1K and PPhOZ–PEI 1K at different compositions. For the remaining copolymers, the results are presented in Figures S1–S4. To determine the hydrolysis ratios of the PEOZ–PEI, PPrOZ–PEI, and PPeOZ–PEI copolymers, peaks at δ_a_ = 3.00–2.65 ppm (4H, −NH–CH_2_–CH_2_−) corresponding to protons of the methylene groups on ethylenimine units and δ_b_ = 3.80–3.28 ppm (4H, −CH_2_–CH_2_–N−) corresponding to protons of the methylene groups on oxazoline units were used. In addition, the peaks corresponding to the methylene and methyl groups of side moieties of PEOZ–PEI were observed at δ_c_ = 2.15–2.35 ppm (2H, −CH_2_) and δ_d_ = 0.98–1.21 ppm (3H, −CH_3_). Hydrogens on side chains of PPrOZ–PEI copolymers show at δ_c_ = 2.22–2.47 ppm (2H, −CH_2_), δ_d_ = 1.50–1.71 ppm (2H, −CH_2_), and δ_e_ = 0.85–1.05 ppm (3H, −CH_3_). In the case of PPeOZ–PEI copolymers, peaks appearing at δ_c_ = 2.13–2.39 ppm (2H, −CH_2_), δ_d_ = 1.43–1.65 ppm (2H, −CH_2_), δ_e_ = 1.43–1.65 ppm (4H, −CH_2_–CH_2_−), and δ_f_ = 0.70–1.02 ppm (3H, −CH_3_) correspond to hydrogen on the side moieties. For PPhOZ–PEI copolymers, the peaks at δ_d_ = 3.6–3.4 ppm (4H, – NH–CH_2_–CH_2_−) corresponding to protons of methylene groups on oxazoline units came together with the CD_3_OD peak seen at δ = 3.30 ppm in a broad peak. Therefore, compositions of PPhOZ–PEI copolymers were calculated using peaks at δ_a_ = 3.00–2.65 ppm (4H, −NH–CH_2_–CH_2_−) and δ_b_ = 7.9–6.9 ppm (5H, phenyl ring). The following equation was used to calculate the hydrolysis ratios of synthesized copolymers based on integral values (*I*)
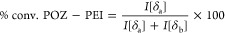


FTIR analysis was also conducted to confirm the structure of the synthesized POZ–PEI copolymers. FTIR spectra of PPeOZ–PEI 1K and PPhOZ–PEI 1K copolymers in all compositions are presented in Figure 2b,d along with the FTIR spectra of PPeOZ 1K and PPhOZ 1K homopolymers. FTIR signals observed at 2870–2976 cm^–1^ corresponded to CH_3_ and CH_2_ vibrations, those at 3280 cm^–1^ corresponded to the NH peak of POZ–PEI, and those at 1196 cm^–1^ corresponded to C–N stretches for all copolymers. The sharp peak observed at 1626 cm^–1^ corresponded to the amide carbonyl (C=O) peak of oxazoline units. It is worth noting that the peak belonging to NH was absent in the POZ homopolymers. The presence of this peak in the spectra of the synthesized POZ–PEI copolymers is an indication that hydrolysis is taking place. In addition, as the hydrolysis ratio increased, the peaks for NH and C–N stretching intensified. In contrast, the opposite trend was observed for the C=O peak.

**Figure 3:**
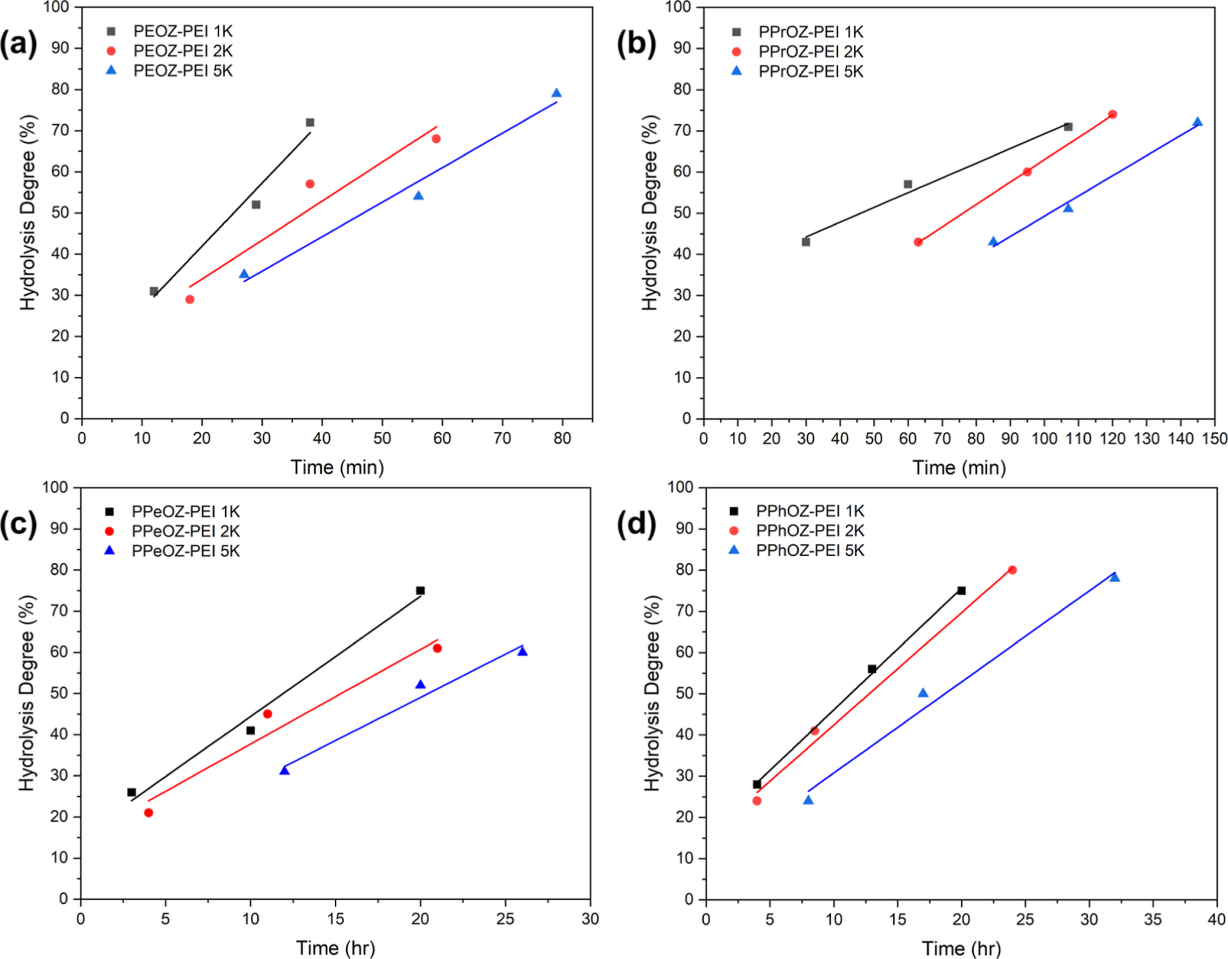
Time versus hydrolysis degree graphs and the corresponding linear regressions (*R*^2^ > 0.93) for PEOZ–PEI (a), PPrOZ–PEI (b), PPeOZ–PEI (c), and PPhOZ–PEI (d) copolymers at all compositions.

Figure 3 shows the compositions of all synthesized copolymers versus their hydrolysis time. Here, the effect of the molar mass of the precursor POZ homopolymers can be observed. Contrary to the reports in the literature, moving from 1K molar mass to 5K, more time was required to reach a specific degree of hydrolysis.^11^ This can be attributed to the oligomeric structure of the homopolymers, which affects the hydrolysis kinetics significantly.

In addition, the effect of the reaction temperature is also evident in the graphs in Figure 3. For the PEOZ and PPrOZ homopolymers, the reaction was performed at 100 °C, whereas for PPeOZ and PPhOZ, the reaction was performed at 85 °C. The hydrolysis advances at a higher rate when temperature is increased, which shows itself in the time that is needed to reach a specific hydrolysis ratio. For PEOZ and PPrOZ, the hydrolysis occurs on a scale of minutes, whereas for PPeOZ and PPhOZ, the same hydrolysis degree is achieved in a matter of hours. In addition, the hydrolysis in ethanol–water mixture is naturally slower than that in pure water. The hydrophobic and bulkiness of pentyl and phenyl also plays a role in the slower hydrolysis rate of PPeOZ and PPhOZ homopolymers.

### 3.2 Thermal Analysis

#### 3.2.1 DSC Results

**Figure 4:**
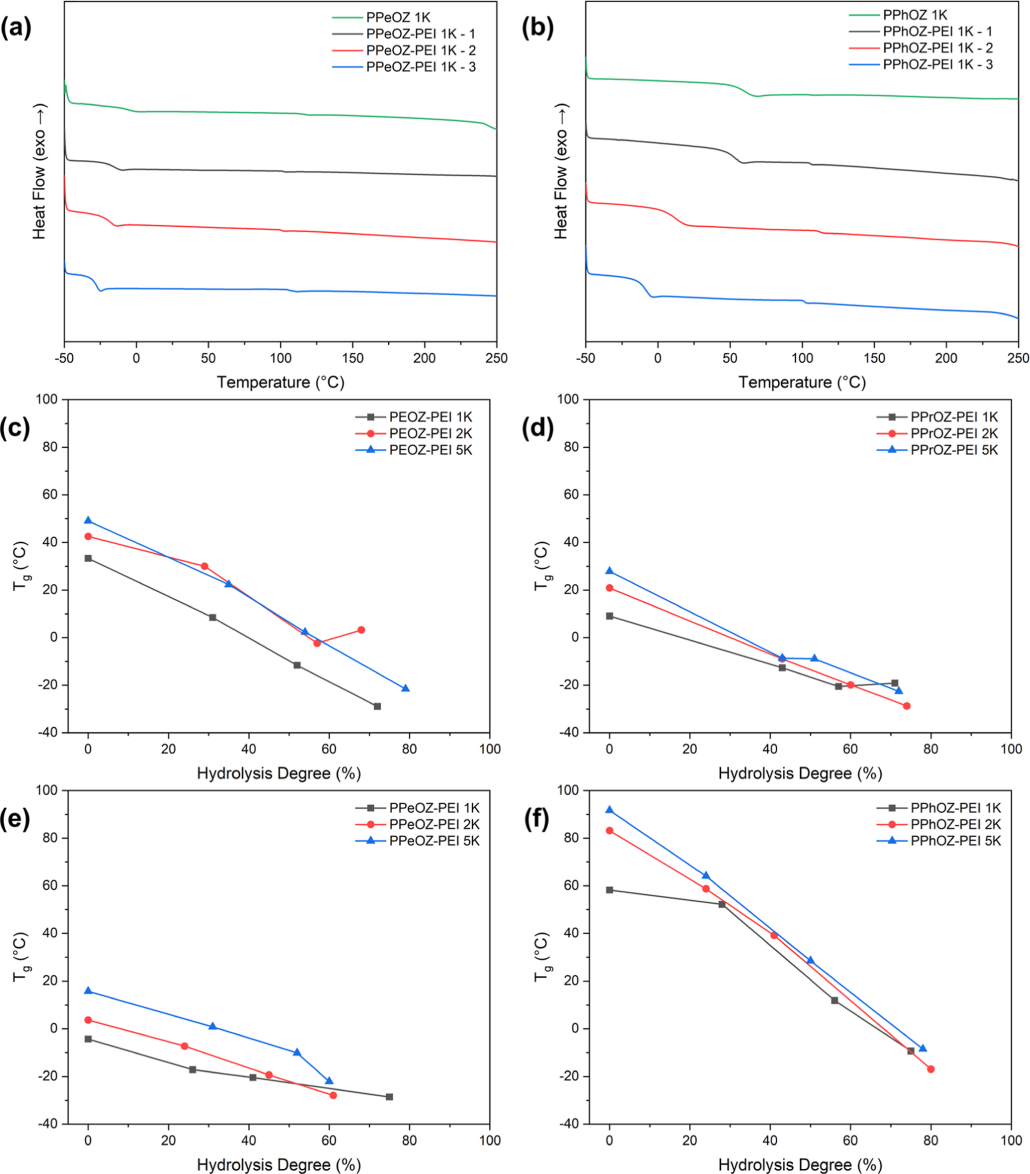
DSC thermograms of (a) PPeOZ–PEI 1K and (b) PPhOZ–PEI 1K at all compositions along with PPeOZ 1K and PPhOZ 1K homopolymers. *T*_g_ values of PEOZ–PEI (c), PPrOZ–PEI (d), PPeOZ–PEI (e), and PPhOZ–PEI (f) copolymers based on their hydrolysis ratios and molar mass. Heating rate: 10 °C/min.

The thermal behavior of the precursor POZ homopolymers and the synthesized POZ–PEI copolymers was investigated by DSC. Figure 4 shows the DSC thermograms of PPeOZ–PEI 1K and PPhOZ–PEI 1K at all compositions along with the *T*_g_ values of all copolymers based on their hydrolysis ratios and molar mass. *T*_g_ values of POZ–PEI copolymers varied between −29 and 64 °C. All POZ–PEI copolymers showed lower *T*_g_ values in comparison to their precursor POZ homopolymers, which is due to the higher flexibility of ethylenimine units compared to that of oxazoline units. For POZ–PEI copolymers with the same pendant groups, *T*_g_ decreased as the hydrolysis ratio increased for all of the molar masses. Also, copolymers with higher molar masses showed higher *T*_g_ values at a constant hydrolysis ratio. For POZ–PEI copolymers with alkyl side chains, as the length of alkyl chains increased, there was a decreasing trend in *T*_g_ values, with PPeOZ–PEI copolymers having the lowest *T*_g_. POZ–PEI copolymers with aryl side chains showed higher *T*_g_ values compared with their alkyl counterparts. Additionally, it was observed that PPeOZ 5K showed a crystalline structure with a *T*_hc_ at 99 °C and a *T*_m_ at 157 °C. This was consistent with the data reported in the literature for the thermal properties of POZ homopolymers with *n*-alkyl pendant groups.^37^ Specifically, for POZ homopolymers with four or more carbons in their pendant groups, side chain crystallization was observed.^37^ For PPeOZ 2K and PPeOZ 1K homopolymers, a semicrystalline and amorphous structure was observed, respectively. This indicated an increase in the crystallinity of PPeOZ homopolymers with an increase in molecular weight. In the case of the PPeOZ–PEI copolymers, the hydrolyzed PPeOZ–PEI 5K samples showed amorphous structure. This can be observed in the PPeOZ–PEI 5K-1 copolymer as *T*_hc_ disappeared (Figure S5). PPeOZ 2K had a semicrystalline structure, and hydrolysis changed the structure of this polymer to amorphous in PPeOZ–PEI 2K-1 copolymer (Figure S5). A low-enthalpy *T*_m_, in comparison to *T*_m_ seen in the PPeOZ 2K homopolymer, was observed without *T*_cc_. This indicates that the crystallinity of the polymers also decreased as hydrolysis proceeded. PEOZ–PEI, PPrOZ–PEI, and PPhOZ–PEI copolymers and their precursor homopolymers all showed amorphous structure. For these polymers, *T*_g_ determined whether they existed as solids or waxy materials at room temperature.

#### 3.2.2 TGA Results

**Figure 5:**
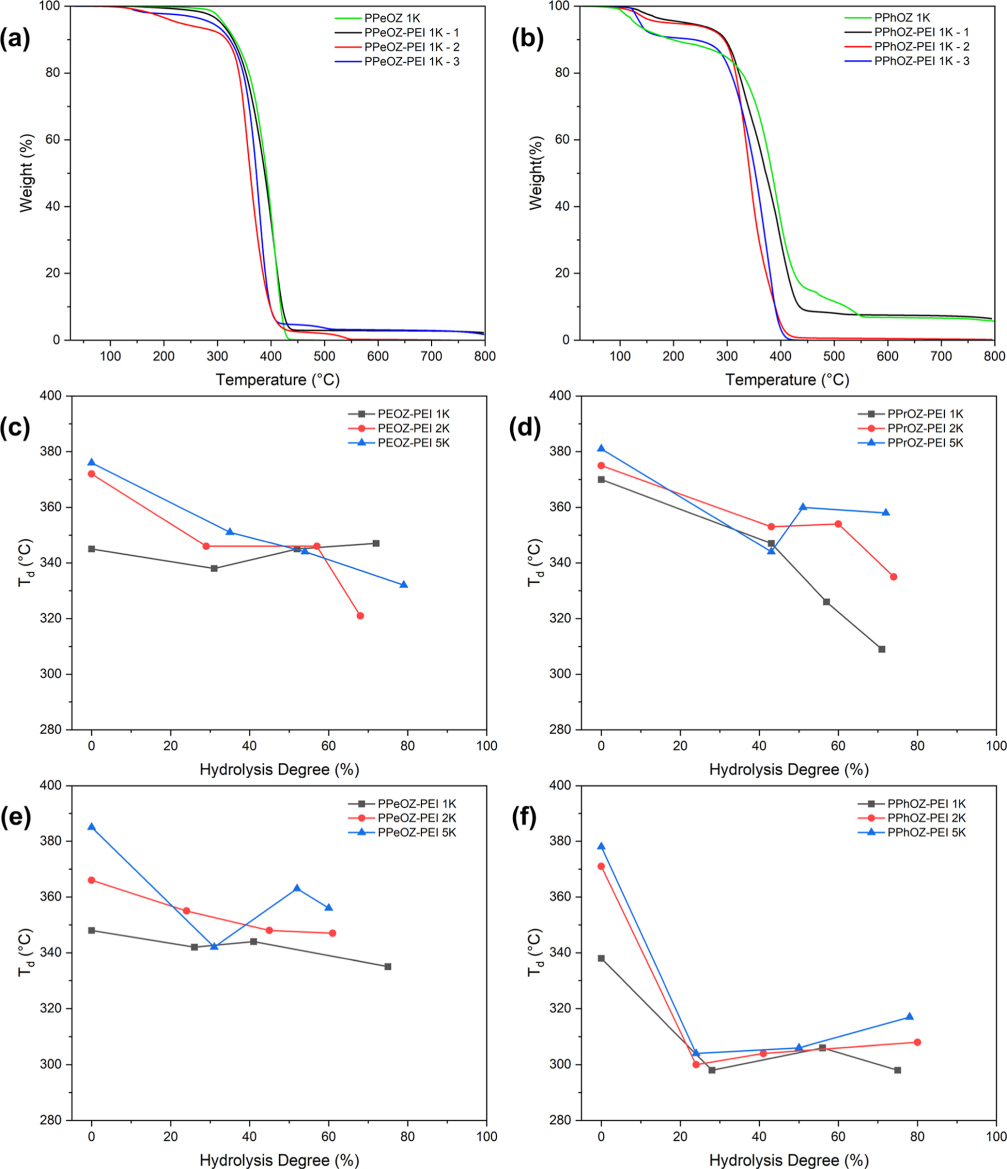
TGA thermograms of the synthesized (a) PPeOZ–PEI 1K and (b) PPhOZ–PEI 1K copolymers at all compositions together with *T*_d_ values of all (c) PEOZ–PEI, (d) PPrOZ–PEI, (e) PPeOZ–PEI, and (f) PPhOZ–PEI copolymers based on the hydrolysis ratio and molar mass.

TGA thermograms of the synthesized PPeOZ–PEI 1K and PPhOZ–PEI 1K copolymers at all compositions together with *T*_d_ values of all copolymers based on hydrolysis ratio and molar mass are presented in Figure 5. The TGA tests were performed at temperatures between 25 and 800 °C. The drops seen between 100 and 200 °C are attributed to presence of water or leftover solvent. All POZ–PEI polymers lost approximately 94.0–99.9% of their weight at 800 °C. This implies that the purification procedure used in the synthesis of these polymers can isolate the product with high purity. POZ–PEI copolymers with alkyl side chains show a one-step decomposition profile, whereas the obtained PPhOZ–PEI copolymers have a multistep decomposition profile at low hydrolysis ratios and a one-step decomposition profile at high hydrolysis ratios. Decomposition temperatures of POZ–PEI copolymers varied between 298 and 363 °C. All POZ–PEI copolymers show lower *T*_d_ values compared to their precursor POZ homopolymers. Moreover, in comparison to POZ–PEI copolymers alkyl side chains, the PPhOZ–PEI copolymers with aryl side chains had a lower decomposition temperature at the same molar mass and hydrolysis ratio. Hydrolysis affected the *T*_d_ values of the POZ–PEI copolymers with alkyl pendant groups differently from the POZ–PEI copolymers with aryl pendant groups. For poly(2-alkyl-2-oxazoline)s, there was a decreasing trend in decomposition temperature with increasing hydrolysis ratio. However, in case of the PPhOZ–PEI copolymers, the lowest decomposition temperature was observed at low degrees of hydrolysis. As hydrolysis progressed, the decomposition temperature also increased. This difference between alkyl and aryl containing POZ–PEI copolymers can be attributed to the different thermal decomposition mechanisms of these polymers. In copolymers with alkyl side chains, it seems that the decomposition starts in the backbone and ethylenimine units show a lower decomposition temperature in comparison to the oxazoline backbone. However, in phenyl-containing homopolymers, the aryl side chains are determining the thermal decomposition temperature. Therefore, with the introduction of ethylenimine units, the decomposition temperature experiences a sharp decrease at low hydrolysis ratios.

### 3.3 Curing Studies

#### 3.3.1 Dynamic DSC Results

The obtained copolymers were mixed at an amine-to-epoxy molar ratio of 1:1 to prepare POZ–PEI copolymer-based OCERs. The calculations, the amount of DGEBA and POZ–PEI TLCs for each OCER, and the amine hydrogen equivalent weight (AHEW) for each TLC are given in Table S4. The effects of molar mass (1K, 2K, and 5K), pendant group (ethyl, propyl, pentyl, and phenyl), and composition (25%, 50%, and 75% hydrolysis degree) on the curing behavior and thermal latency of the prepared OCERs were investigated. Table 1 shows the dynamic DSC results of the prepared OCERs. The left limit temperatures of TLCs ranged between 38.56 and 111.22 °C. The observed normalized curing enthalpy values of OCERs varied between 8.66 and 198.33 J/g. As can been seen from the data, there was an inverse relation between the enthalpy of curing and the left limit temperatures. The lowest left limit temperature and highest enthalpy of curing were observed in OCERs with PEOZ–PEI 5K-3 TLCs. The opposite, the highest left limit temperature and the lowest enthalpy of curing, was seen in OCER with PPeOZ–PEI 2K-2 TLC.

**Table 1:** Dynamic DSC Results of the OCERs Containing POZ–PEI TLCs

copolymer-DGEBA	hydrolysis degree (%)	normalized curing enthalpy (J/g)	left limit temperature (°C)	right limit temperature (°C)
PEOZ–PEI 1K-1-DGEBA	31	45.22	78.11	174.78
PEOZ–PEI 1K-2-DGEBA	52	118.11	49.43	172.44
PEOZ–PEI 1K-3-DGEBA	72	185.85	45.69	166.06
PEOZ–PEI 2K-1-DGEBA	29	48.49	85.65	197.10
PEOZ–PEI 2K-2-DGEBA	57	69.67	67.83	176.72
PEOZ–PEI 2K-3-DGEBA	68	25.44	85.25	170.88
PEOZ–PEI 5K-1-DGEBA	35	26.50	82.61	132.61
PEOZ–PEI 5K-2-DGEBA	54	81.86	55.93	173.54
PEOZ–PEI 5K-3-DGEBA	79	198.33	38.56	176.58
PPrOZ–PEI 1K-1-DGEBA	43	92.72	47.48	168.39
PPrOZ–PEI 1K-2-DGEBA	57	109.08	55.58	171.92
PPrOZ–PEI 1K-3-DGEBA	71	50.65	60.94	181.64
PPrOZ–PEI 2K-1-DGEBA	43	78.67	65.88	187.82
PPrOZ–PEI 2K-2-DGEBA	60	164.08	42.19	186.64
PPrOZ–PEI 2K-3-DGEBA	74	155.97	52.02	188.94
PPrOZ–PEI 5K-1-DGEBA	43	188.02	43.54	198.05
PPrOZ–PEI 5K-2-DGEBA	51	96.96	68.31	199.66
PPrOZ–PEI 5K-3-DGEBA	72	117.49	59.05	178.89
PPeOZ–PEI 1K-1-DGEBA	26	23.54	86.37	199.26
PPeOZ–PEI 1K-2-DGEBA	41	10.66	109.30	199.87
PPeOZ–PEI 1K-3-DGEBA	75	174.88	42.77	170.60
PPeOZ–PEI 2K-1-DGEBA	21	59.66	109.95	199.50
PPeOZ–PEI 2K-2-DGEBA	45	8.66	111.22	198.16
PPeOZ–PEI 2K-3-DGEBA	61	61.96	68.77	181.47
PPeOZ–PEI 5K-1-DGEBA	31	46.02	83.05	200.00
PPeOZ–PEI 5K-2-DGEBA	52	131.93	62.25	198.87
PPeOZ–PEI 5K-3-DGEBA	60	146.55	62.93	185.41
PPhOZ–PEI 1K-1-DGEBA	28	37.01	92.39	197.63
PPhOZ–PEI 1K-2-DGEBA	56	51.12	83.47	179.63
PPhOZ–PEI 1K-3-DGEBA	75	102.71	51.70	173.97
PPhOZ–PEI 2K-1-DGEBA	24	44.32	72.98	199.19
PPhOZ–PEI 2K-2-DGEBA	41	26.96	100.13	198.17
PPhOZ–PEI 2K-3-DGEBA	80	152.06	47.62	173.81
PPhOZ–PEI 5K-1-DGEBA	24	24.18	93.54	181.56
PPhOZ–PEI 5K-2-DGEBA	50	16.41	104.98	193.26
PPhOZ–PEI 5K-3-DGEBA	78	93.32	61.78	172.65

##### 3.3.1.1 Effect of Composition on the Curing Behavior of POZ–PEI-Based OCERs

**Figure 6:**
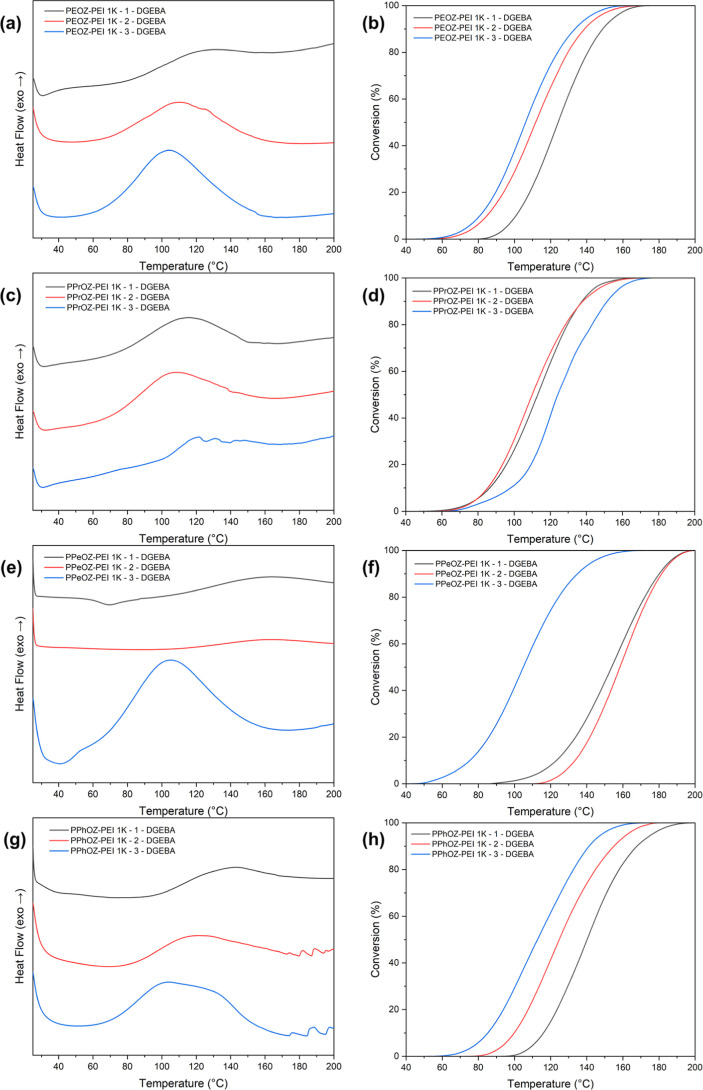
Curing and conversion curves for DGEBA with PEOZ–PEI (a,b), PPrOZ–PEI (c,d), PPeOZ–PEI (e,f), and PPhOZ–PEI (g,h) 1K TLCs at all compositions.

For the prepared POZ–PEI-based OCERs, no universal trend is evident between the composition and curing behavior. However, certain patterns are observed. For instance, for OCERs with PEOZ–PEI 1K, PEOZ–PEI 5K, PPeOZ–PEI 5K, and PPhOZ–PEI 1K TLCs, the hydrolysis ratio has a direct relation with normalized curing enthalpy, alongside an inverse relation with left limit temperature. This is reflected in the curing and conversion curves of the mentioned TLCs as a shift of exothermic curing peak to lower temperatures with the increase of hydrolysis ratio. The curing and conversion curves for POZ–PEI 1K are presented in Figure 6. The curing data for POZ–PEI 2K and 5K are also shown in Figures S6 and S7. However, this trend does not hold for all samples, likely due to the interplay of multiple factors. The hydrophilicity/hydrophobicity of the pendant groups, bulkiness of the side chains, and number of amine groups act synergistically or antagonistically at low to mild hydrolysis ratios. In addition, differences in *T*_g_ of the copolymers further influence the curing behavior. For TLCs with *T*_g_ values above room temperature, aggregation during mixing can lead to uneven temperature distribution and nonsmooth curing profiles. Generally, TLCs with high hydrolysis ratios show the lowest left limit temperatures and highest normalized curing enthalpy in comparison to TLCs with the same molar mass of the precursor homopolymer and the same pendant groups. At high hydrolysis ratios, the effect of pendant groups begins to diminish, which in turn promotes a better accessibility of DGEBA to the amine groups. In contrast, at low to mild hydrolysis ratios, with the presence of a higher number of amide groups, the hydrogen bonding between these groups and the hydroxyl/ether groups of DGEBA can affect the curing reaction. In other words, this hydrogen bonding can slow down the reaction between epoxides and secondary amine groups, resulting in irregular curing behavior. It is worth noting that for the PPrOZ–PEI TLCs, the observed left limit temperatures depending on the compositions vary between 42 and 68 °C, whereas for PEOZ–PEI, PPeOZ–PEI, and PPhOZ–PEI TLCs, a wider interval of left limit temperatures is seen. This can be attributed to the slightly higher hydrolysis ratios of PPrOZ–PEI 1K-1, PPrOZ–PEI 2K-1, and PPrOZ–PEI 5K-1 TLCs compared to those of their counterparts. Among the POZ–PEI TLCs, the best latency considering their left limit temperatures, conversion curves, and enthalpy of curing was observed for the PPhOZ–PEI 1K-1 and PPeOZ–PEI 2K-1 TLCs.

##### 3.3.1.2 Effect of Molar Mass on the Curing Behavior of POZ–PEI-Based OCERs

**Figure 7:**
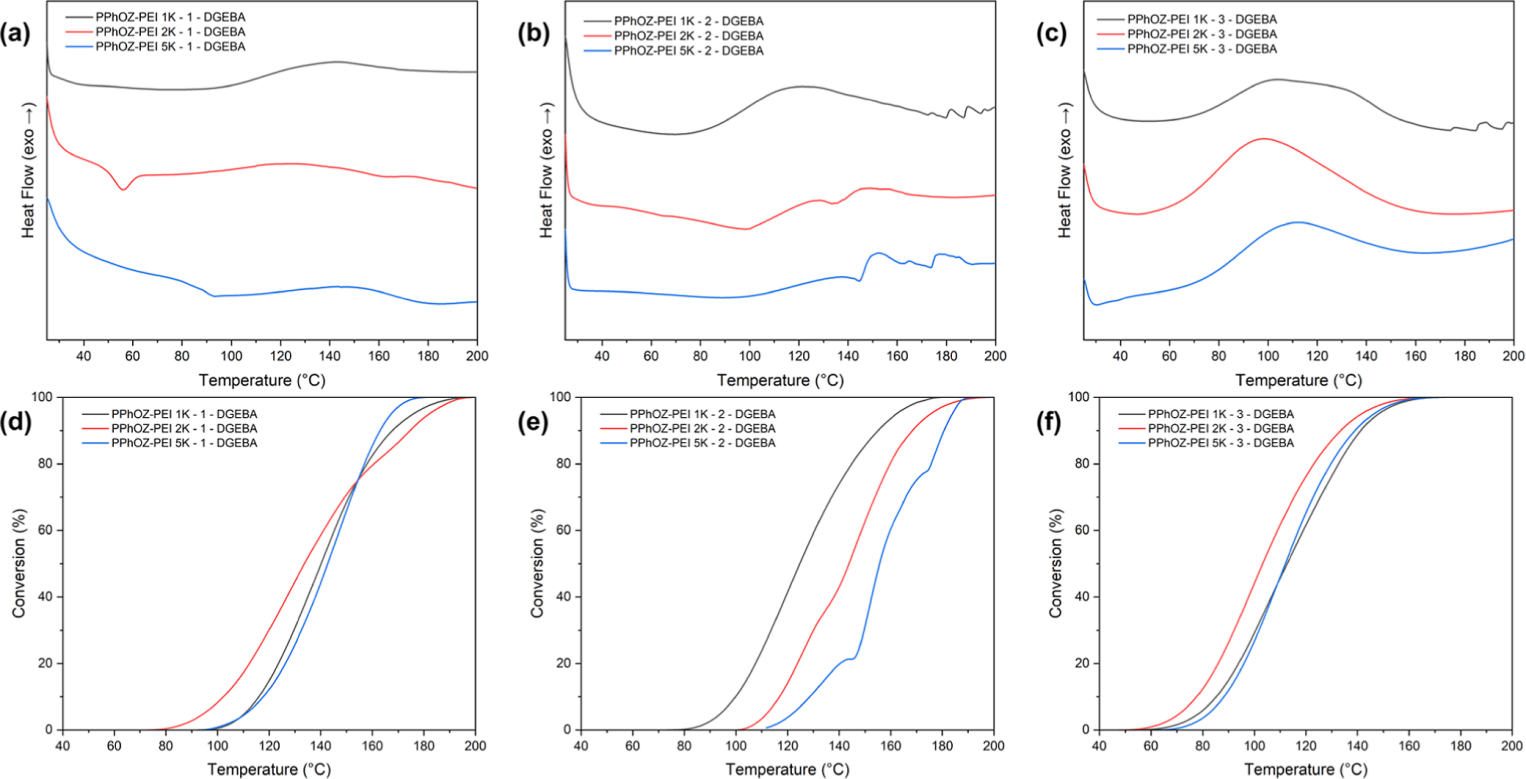
Curing and conversion curves for DGEBA with PPhOZ–PEI TLCs at (a,d) low, (b,e) mild, and (c,f) high hydrolysis ratios based on molecular mass.

Investigation of the effect of molar mass showed that for the POZ–PEI TLCs with the same hydrolysis ratios and pendant groups, increasing the molar mass of the precursor homopolymers did not have a noticeable impact on their curing behavior. It may be due to dominance of steric effects as latency mechanism in POZ–PEI TLCs, which do not change drastically with the increase of molar mass from 1K to 5K at the same compositions and pendant groups. In the case of PPhOZ–PEI TLCs, given that the hydrolysis ratio stays the same, there is only a variance of 20 °C in the left limit temperature with the increase of molar mass. For the PPhOZ–PEI TLCs with DGEBA, the heat flow versus temperature and conversion curves based on different molar masses are shown in Figure 7. Looking at the DSC thermograms and conversion curves of PPhOZ–PEI TLCs, the increase in the molar mass changes the ease of cross-linking rather than the overall curing behavior of TLCs. For PEOZ–PEI, PPrOZ–PEI, and PPeOZ–PEI TLCs also, the change in the left limit temperatures with respect to molar mass does not exceed 20 °C, except for the PEOZ–PEI TLCs at high hydrolysis ratios and PPeOZ–PEI TLCs at mild hydrolysis ratios where a 50 °C variance is observed.

##### 3.3.1.3 Effect of Pendant Groups on the Curing Behavior of POZ–PEI-Based OCERs

Moving from hydrophilic pendant groups to more hydrophobic pendant groups, there is a general trend of increasing the left limit temperatures and decreasing the curing enthalpy. TLCs with pentyl and phenyl pendant groups perform better in comparison to TLCs with ethyl and propyl pendant groups in terms of latency. As we move toward more hydrophobic side chains such as phenyl and pentyl, there are more pronounced steric effects, which in turn decrease the reactivity of the amine groups. This increases the left limit temperature of curing, allowing the amine groups to be activated at elevated temperatures. The pendant groups’ effect is more evident at low to mild hydrolysis ratios. For instance, comparing the PEOZ–PEI 5K and PPhOZ–PEI 5K at mild hydrolysis ratios, there is a 50 °C difference in the left limit temperature and 70 J/g difference in the normalized curing enthalpy. The best latency was also observed for PPeOZ–PEI 2K at a mild hydrolysis ratio with a left limit temperature of 111.22 °C and a normalized curing enthalpy of 8.66 J/g. At high hydrolysis ratios, almost all of the POZ–PEI TLCs show a left limit temperature between 40 and 60 °C. This is due to the low number of oxazoline units in comparison to ethylenimine units at high hydrolysis ratios, which decrease the effect of pendant groups, which in turn shows itself as a convergence in curing behavior of TLCs.

#### 3.3.2 Isothermal DSC Results

**Figure 8:**
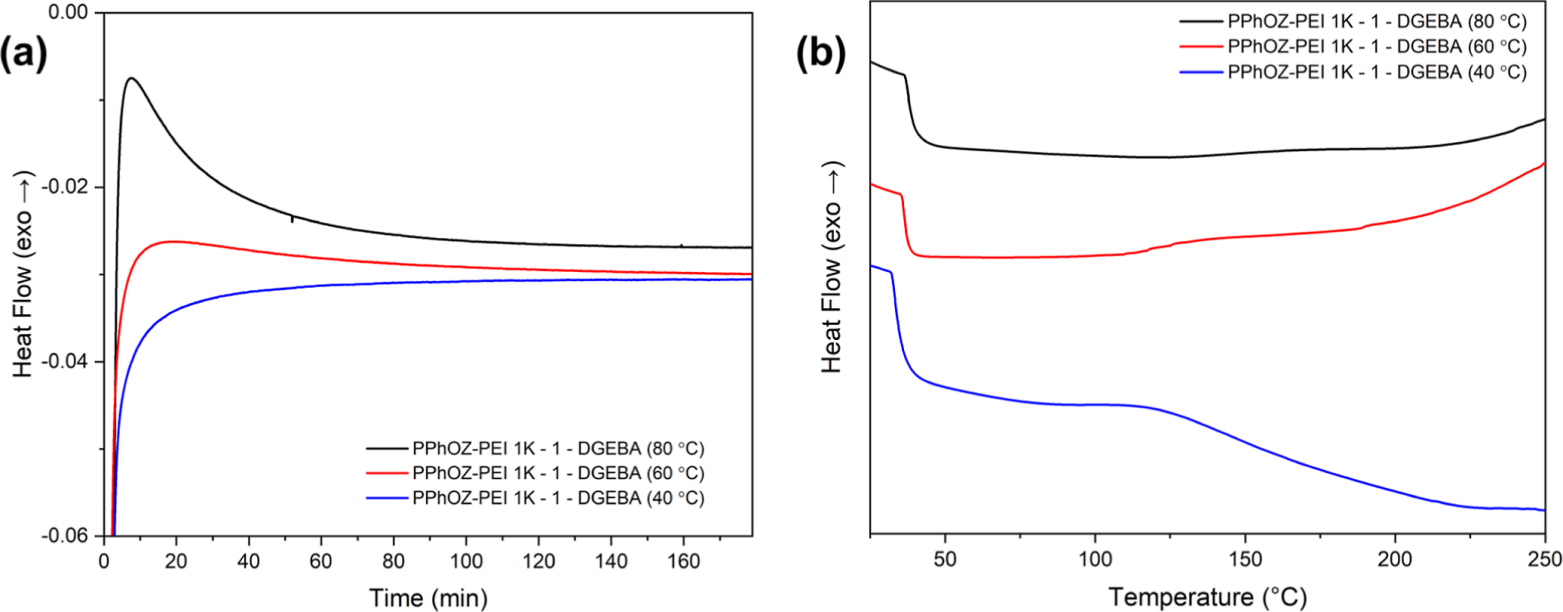
(a) Isothermal DSC and (b) dynamic DSC enthalpy curves for PPhOZ–PEI 1K-1.

Isothermal DSC analyses were performed to test the stability of the best POZ–PEI TLC sample. PPhOZ–PEI 1K-1 was chosen as the best candidate based on its curing enthalpy, left limit temperature, conversion curve, and ease of synthesis. Additionally, this TLC was preferred because its high *T*_g_ can also utilize the physical mechanism besides the chemical route to induce latency. The PPhOZ–PEI 1K-1 copolymer was mixed with DGEBA in a 1:1 epoxy group to –NH ratio in a DSC pan. Isothermal DSC tests were performed at three different temperatures (40, 60, and 80 °C) for 3 h to study the stability of the chosen OCER. After isothermal DSC test, dynamic DSC test was performed on the sample, heating it from 25 to 200 °C at a heating rate of 10 °C/min under a nitrogen atmosphere. Dynamic DSC testing was performed to obtain the residual enthalpy of the sample after the isothermal DSC test. Isothermal and dynamic DSC enthalpy curves are shown in Figure 8. The normalized enthalpy values for the isothermal tests and dynamic DSC tests are presented in Table 2.

**Table 2:** Curing Values of PPhOZ–PEI 1K-1 Isothermal and Dynamic DSC Analyses

polymer	isothermal temperature (°C)	normalized curing enthalpy (J/g)	residual enthalpy (J/g)
PPhOZ–PEI 1K-1-DGEBA	40		55.73
PPhOZ–PEI 1K-1-DGEBA	60	13.49	10.23
PPhOZ–PEI 1K-1-DGEBA	80	37.85	3.64

As can be seen from the isothermal DSC curves and their corresponding enthalpy values, at 40 °C, PPhOZ–PEI 1K-1 was stable, with no curing observed during the isothermal test. The residual enthalpy also shows full curing with an enthalpy of 55.73 J/g, which shows that no curing reaction occurred during the isothermal DSC test. For isothermal tests performed at 60 and 80 °C, curing occurred with enthalpies of 13.49 and 37.85 J/g. However, the residual enthalpy observed in the 60 °C sample was greater than that observed at 80 °C. This shows that the sample has better stability at 60 °C than at 80 °C. It is also worth noting that the left limit temperature seen in the dynamic DSC test for PPhOZ–PEI 1K-1 was 92 °C. The observed difference between the dynamic DSC analysis and isothermal test may be caused by the kinetics of the reaction. This implies that by reducing the heating rate in the dynamic DSC test, the observed left limit temperature may decrease. Thus, PPhOZ–PEI 1K-1 shows full stability at 40 °C and room temperature. This means that this TLC can be utilized to increase the shelf life of OCERs.

### 3.4 Rheology Studies

**Figure 9:**
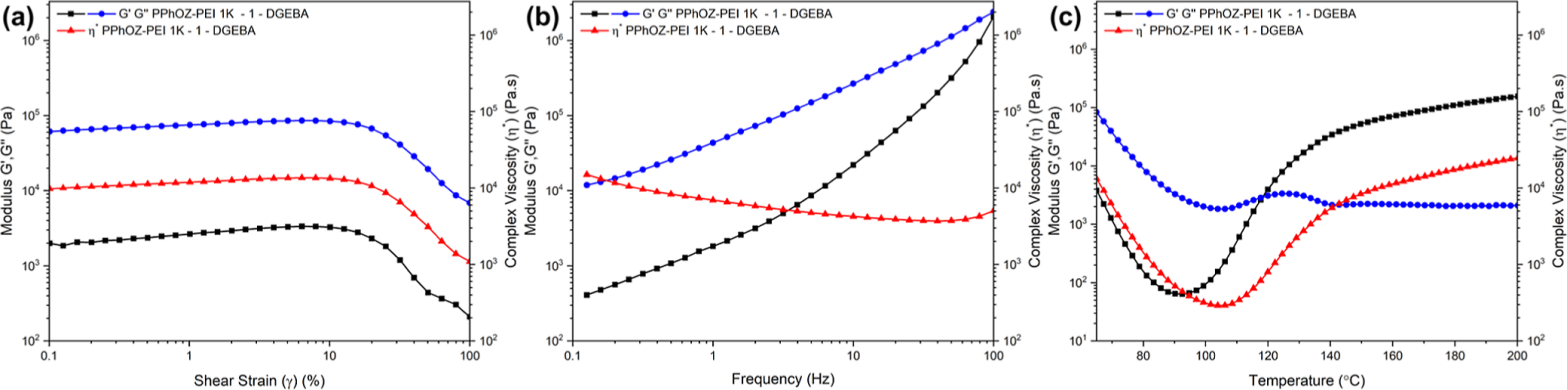
Storage modulus (*G*′), loss modulus (*G*″), and the complex viscosity (η*) of the PPhOZ–PEI 1K-1-DGEBA OCER with respect to shear strain (a), frequency (b), and temperature (c).

To investigate the viscoelasticity, dispersion quality, and curing profile of the PPhOZ–PEI 1K-1-DGEBA OCER, rheology studies were conducted. Due to the high solid content of the sample (70% copolymer w/w), the amplitude and frequency sweeps tests were performed at 65 °C, which was 10 °C higher than the *T*_g_ of the PPhOZ–PEI 1K-1 TLC. Also, the temperature sweep test was conducted between 65 and 200 °C. The complex viscosity (η*), storage modulus (*G*′), and loss modulus (*G*″) of the PPhOZ–PEI 1K-1-DGEBA OCER with respect to shear strain, frequency, and temperature are shown in Figure 9. As shown in Figure 9a, the linear viscoelastic region of the sample extended up to 10% of shear strain, and above this value, *G*′, *G*″, and η* started to decrease and showed dependency on shear strain. The slight increase seen in viscoelastic parameters can be attributed to the onset of curing at 65 °C. In addition, the sample showed viscoelastic fluid-type behavior since *G*″ of the sample stayed higher than *G*′ at all shear strain values. Based on the results of the amplitude sweep, frequency and temperature sweep were conducted at 1% shear strain to ensure that the sample remained in its viscoelastic linear region. In the frequency sweep test (Figure 9b), *G*″ dominated over *G*′ with η* decreasing through the frequency range, which is typical of viscoelastic liquids. However, a crossover was seen at 100 Hz, which shows a transformation to solid-like behavior at high frequencies. This behavior indicates that the viscoelastic fluid-like behavior of the PPhOZ–PEI 1K-1-DGEBA at low frequencies facilitates the mixing until a stable network establishes at high frequencies. Figure 9c shows the changes in viscoelastic parameters with respect to the temperature. A relatively high η* of 13.2 KPa s was observed at 65 °C due to the high amount of the PPhOZ–PEI 1K-1 TLC in the OCER. The minimum η* was 0.2 Kpa, which was observed at 104 °C. Also, *G*″ reached its minimum value, 63.7 Pa, at 93 °C and started elevating afterward. Additionally, a crossover point was seen between *G*′ and *G*″ at 118 °C, which is an indicator of curing taking place and a transformation from fluid-like to solid-like behavior. The η* reached a plateau as the temperature increased and the cured sample showed a maximum η* of 20.5 KPa s and a maximum *G*′ of 128.8 KPa at 200 °C.

### 3.5 Thermomechanical Properties

**Figure 10:**
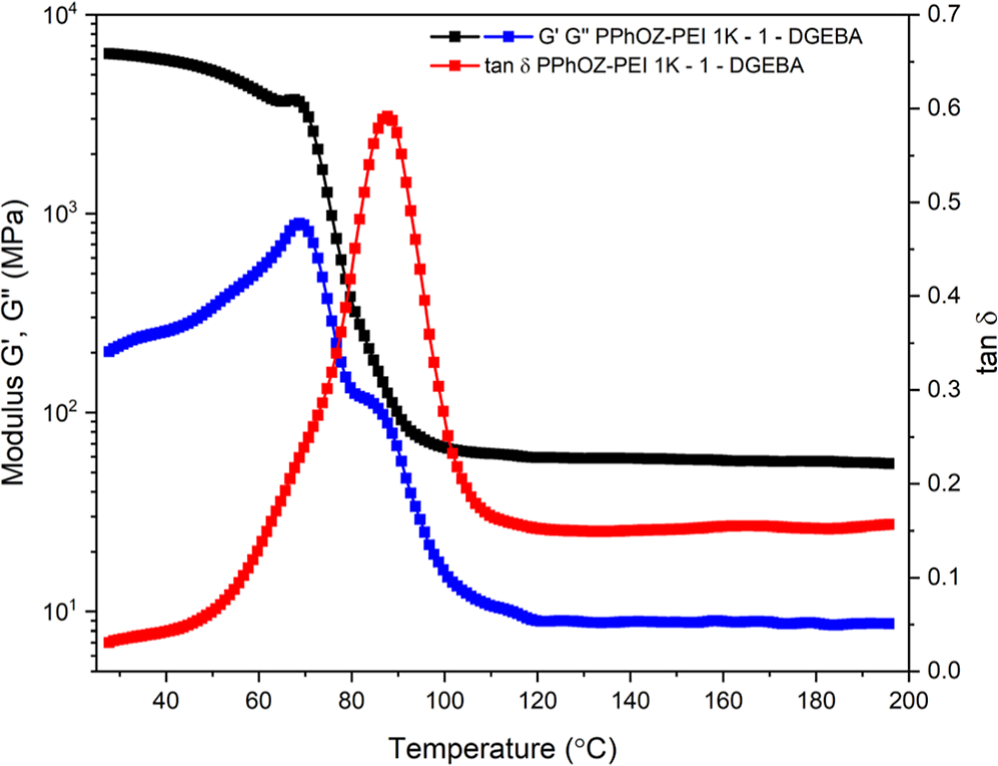
Storage modulus (*G*′), loss modulus (*G*″), and tan δ of the cured PPhOZ–PEI 1K-1-DGEBA OCER with respect to temperature.

DMA analysis was performed to determine the thermomechanical properties of the PPhOZ–PEI 1K-1-DGEBA OCER after curing in terms of storage modulus (*G*′), loss modulus(*G*″), and tan δ (Figure 10). The analysis was conducted from 25 to 200 °C with a heating rate of 3 °C/min. At 25 °C, the sample showed a *G*′ value of 6.3 GPa and a *G*″ value of 0.2 GPa. As the temperature increased, *G*′ started to decrease, while *G*″ showed an initial increase and a subsequent decrease. The onset values of drop for *G*′ and *G*″ were 70.7 and 63.6 °C, respectively. Tan δ showed a peak at 87.6 °C, which was taken as the *T*_g_ of the cured sample. After around 90 °C, *G*′ stayed almost constant with a value of 55.4 MPa at 200 °C. The same behavior was also observed with G″,where the value reached a plateau after 110 °C with a value of 8.6 MPa at 200 °C.

## 4 Conclusions

POZ homopolymers with different alkyl and aryl pendant groups at three molar masses were prepared. These homopolymers were hydrolyzed at three hydrolysis ratios of between 25% and 75%. Effect of hydrolysis ratio, molar mass, and pendant groups on the thermal properties of POZ–PEI copolymers were investigated and compared with that of POZ homopolymers. All POZ–PEI copolymers showed lower *T*_g_ values in comparison to their precursor homopolymers. As the degree of hydrolysis increased, the *T*_g_ values decreased. For PPeOZ 5K and PPeOZ 2K, which have crystalline and semicrystalline structures, hydrolysis changed the structure of the resulting PPeOZ–PEI copolymers to amorphous. In addition, *T*_d_ values of the POZ–PEI copolymers were lower than those of the precursor homopolymer. For POZ–PEI polymers with alkyl side chains, the decomposition temperature decreased as the degree of hydrolysis increased. However, for the PPhOZ–PEI copolymers, there was a sharp decrease in the decomposition temperature at low hydrolysis degrees, and as the hydrolysis progressed, *T*_d_ increased slightly. For POZ polymers with alkyl side chains, TGA results showed a one-step degradation, whereas for PPhOZ–PEI copolymers with low hydrolysis ratios, there was a multistep degradation, and as the ratio of hydrolysis increased, it changed to a one-step degradation curve. The synthesized POZ–PEI copolymers were utilized as TLCs for the preparation of OCERs. The results confirmed that the prepared TLCs provide latency based on their molar mass, side chain groups, and hydrolysis ratio. In contrast to molar mass, the composition and pendant group type proved to have strong impact on the curing behavior of the POZ–PEI TLCs. The TLCs with more hydrophobic pendant groups, PPeOZ–PEI and PPhOZ–PEI TLCs, provided the highest latency in the curing studies due to steric effects. Additionally, the molar mass did not have a noticeable impact on the curing behavior of the POZ–PEI TLCs. Composition showed no clear trend on the curing behavior of TLCs at low to mild hydrolysis ratios. However, at high hydrolysis ratios, with the decrease in the number of oxazoline groups, TLCs showed convergence in curing behavior with left limit temperatures varying between 40 and 60 °C. PPhOZ–PEI 1K-1 was chosen as the best sample based on its left limit temperature, curing enthalpy, and ease of synthesis. Isothermal DSC tests were performed on this sample to investigate its stability. The chosen sample was shown to be stable at 40 °C for 3 h; therefore, it is suitable for use in OCERs at room temperature. The rheology studies showed that the chosen OCER had a linear viscoelastic region ranging up to 10% shear strain. In addition, at high frequencies, a transformation from viscoelastic-fluid to viscoelastic solid due to the compatibility of the PPhOZ–PEI 1K-1 with DGEBA was observed. The curing profile of the chosen sample was studied by temperature sweep rheology, which showed a crossover temperature of 118 °C. The DMA results also showed the evolution of storage and loss moduli and tan δ of the cured sample with respect to temperature. tan δ showed a peak at 87.6 °C, which was taken as the *T*_g_ of the cured PPhOZ–PEI 1K DGEBA OCER.

This research demonstrates the potential of POZ–PEI copolymers as a versatile platform for tailorable curing agents with a wide range of properties and curing behaviors. The investigated relation between curing behavior and structural parameters paves the way for developing curing agents with the desired properties. Nevertheless, further research is required to enhance the mechanical properties of cured epoxy resins. Future studies could explore the integration of additional curing agents into the structure of these copolymers, utilization of cocuring agents, and various postpolymerization modifications to address these challenges. These approaches may expand the functionality and performance of POZ–PEI-based curing systems, making them suitable for advanced composite applications.

## Supporting Information Available

Synthesis conditions of homopolymers, characterization results of homopolymers, ^1^H NMR and FTIR spectra of copolymers, DSC thermograms of copolymers, compositions of prepared OCERs, and the curing data of TLCs (PDF)
