# Supplementary material for: Partially Hydrolyzed Poly(2-alkyl/aryl-2-oxazoline)s as Thermal Latent Curing Agents: Effect of Composition and Pendant Groups on Curing Behavior
Source: ACS Omega. 2025 Feb 14;10(7):6753–67. doi: 10.1021/acsomega.4c08659 (PMC11866178; doi:10.1021/acsomega.4c08659)
Supplement: Supplementary file 1 — ao4c08659_si_002.pdf [file ao4c08659_si_002.pdf]

Supporting Information

Partially Hydrolyzed Poly(2-Alkyl/Aryl-2-Oxazoline)s as Thermal Latent Curing Agents: Effect of Composition and Pendant Groups on Curing Behavior

Saeed Salamatgharamaleki[1,2], Asu Ece Atespare[1, 2], Taha Behroozi Kohlan[1, 2, ‡], Mehmet Yildiz[1,2], Yusuf Ziya Menceloglu[1, 2], Serkan Unal[1, 2], Bekir Dizman[1, 2] *

[1] Integrated Manufacturing Technologies Research and Application Center & Composite Technologies Center of Excellence, Sabanci University, Istanbul, Turkey, 34906

[2] Faculty of Engineering and Natural Sciences, Materials Science and Nano Engineering, Sabanci University, Istanbul, Turkey, 34956

‡ Present address: Department of Fibre and Polymer Technology, KTH Royal Institute of Technology, Stockholm, Sweden

*Corresponding author: Bekir Dizman (bekirdizman@sabanciuniv.edu)

1. Homopolymer and Copolymer Synthesis and Characterization

1.1. Polymerization Temperature and Time for POZ Homopolymers

Table S1. Polymerization temperature and duration for PEOZ, PPrOZ, PPeOZ, and PPhOZ homopolymers

Homopolymers	Temperature (°C)	Polymerization Time (min)
**PEOZ**	80	30, 60, and 120 for 1, 2, and 5 Kg/mol, respectively
**PPrOZ**	80	30, 60, and 120 for 1, 2, and 5 Kg/mol, respectively
**PPeOZ**	80	60, 120, and 240 for 1, 2, and 5 Kg/mol, respectively
**PPhOZ**	95	60, 120, and 240 for 1, 2, and 5 Kg/mol, respectively

1.2. SEC Results of the POZ Homopolymers

Table S2. SEC results of POZ homopolymers and reaction yields.

Polymer	SEC						Yield (%)
	PEOZ Std			PMMA Std			
	M_p (Da)	M_n (Da)	Đ	M_p (Da)	M_n (Da)	Đ	
**PEOZ 1K**	900	700	1.15	1,700	1,200	1.39	81
**PEOZ 2K**	2,500	2,100	1.13	5,400	4,400	1.15	83
**PEOZ 5K**	4,400	3,200	1.22	9,100	6,800	1.18	80
**PPrOZ 1K**	800	700	1.32	1,700	1,100	1.67	84
**PPrOZ 2K**	1,600	1,500	1.16	3,500	2,500	1.33	83
**PPrOZ 5K**	3,700	3,300	1.11	7,700	7,700	1.09	88
**PPeOZ 1K**	800	700	1.13	1,500	1,100	1.35	66
**PPeOZ 2K**	1,500	1,300	1.19	3,000	2,500	1.25	60
**PPeOZ 5K**	3,300	2,300	1.23	6,500	4,600	1.25	83
**PPhOZ 1K**	1,600	1000	1.36	2,600	1,400	1.64	79
**PPhOZ 2K**	1,900	1,500	1.22	3,400	2,500	1.23	81
**PPhOZ 5K**	4,600	4,100	1.08	9,400	8,600	1.06	85

1.3. Hydrolysis Temperature and Time for POZ-PEI Copolymers

Table S3. Hydrolysis Temperature and Time for POZ-PEI Copolymers

Copolymer	Hydrolysis Duration (min)	Copolymer	Hydrolysis Duration (h)
**PEOZ-PEI 1K - 1**	12	**PPeOZ-PEI 1K - 1**	3
**PEOZ-PEI 1K - 2**	29	**PPeOZ-PEI 1K - 2**	15
**PEOZ-PEI 1K - 3**	38	**PPeOZ-PEI 1K - 3**	20
**PEOZ-PEI 2K - 1**	18	**PPeOZ-PEI 2K - 1**	4
**PEOZ-PEI 2K - 2**	38	**PPeOZ-PEI 2K - 2**	11
**PEOZ-PEI 2K - 3**	59	**PPeOZ-PEI 2K - 3**	21
**PEOZ-PEI 5K - 1**	27	**PPeOZ-PEI 5K - 1**	12
**PEOZ-PEI 5K - 2**	56	**PPeOZ-PEI 5K - 2**	20
**PEOZ-PEI 5K - 3**	79	**PPeOZ-PEI 5K - 3**	26
**PPrOZ-PEI 1K - 1**	30	**PPhOZ-PEI 1K - 1**	4
**PPrOZ-PEI 1K - 2**	60	**PPhOZ-PEI 1K - 2**	13
**PPrOZ-PEI 1K - 3**	107	**PPhOZ-PEI 1K - 3**	20
**PPrOZ-PEI 2K - 1**	63	**PPhOZ-PEI 2K - 1**	4
**PPrOZ-PEI 2K - 2**	95	**PPhOZ-PEI 2K - 2**	8.5
**PPrOZ-PEI 2K - 3**	120	**PPhOZ-PEI 2K - 3**	24
**PPrOZ-PEI 5K - 1**	85	**PPhOZ-PEI 5K - 1**	8
**PPrOZ-PEI 5K - 2**	107	**PPhOZ-PEI 5K - 2**	17
**PPrOZ-PEI 5K - 3**	145	**PPhOZ-PEI 5K - 3**	32

2. ^{1}H NMR and FTIR Spectra of Synthesized Copolymers

2.1. ^{1}H NMR and FTIR Spectra of PEOZ-PEI Copolymers

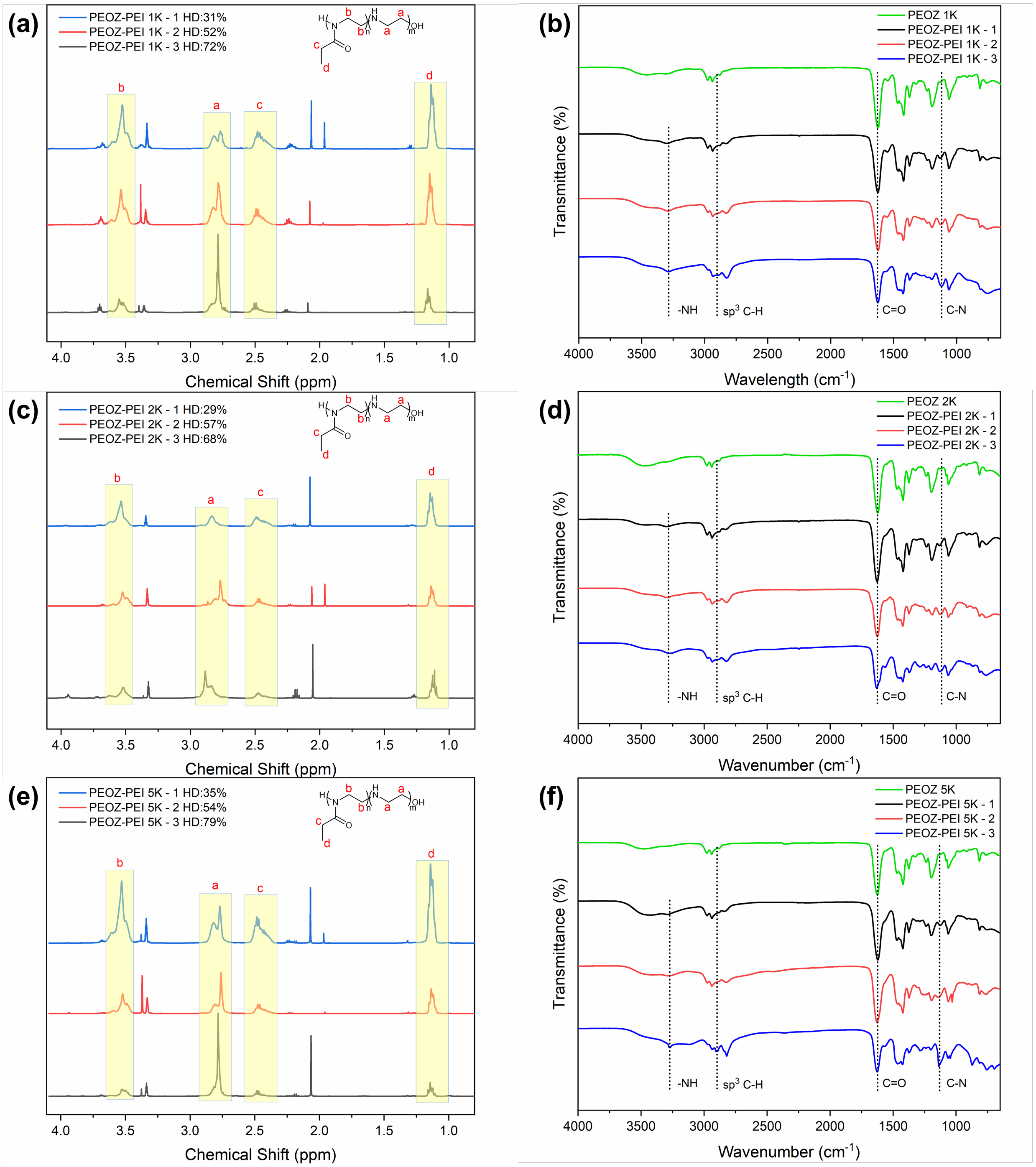

Figure S1. The ^{1}H NMR and FTIR Spectra of (a, b) PEOZ-PEI 1K, (c,d) PEOZ-PEI 2K, and (e, f) PEOZ-PEI 5K Copolymers

2.2. ^{1}H NMR and FTIR Spectra of PPrOZ-PEI Copolymers

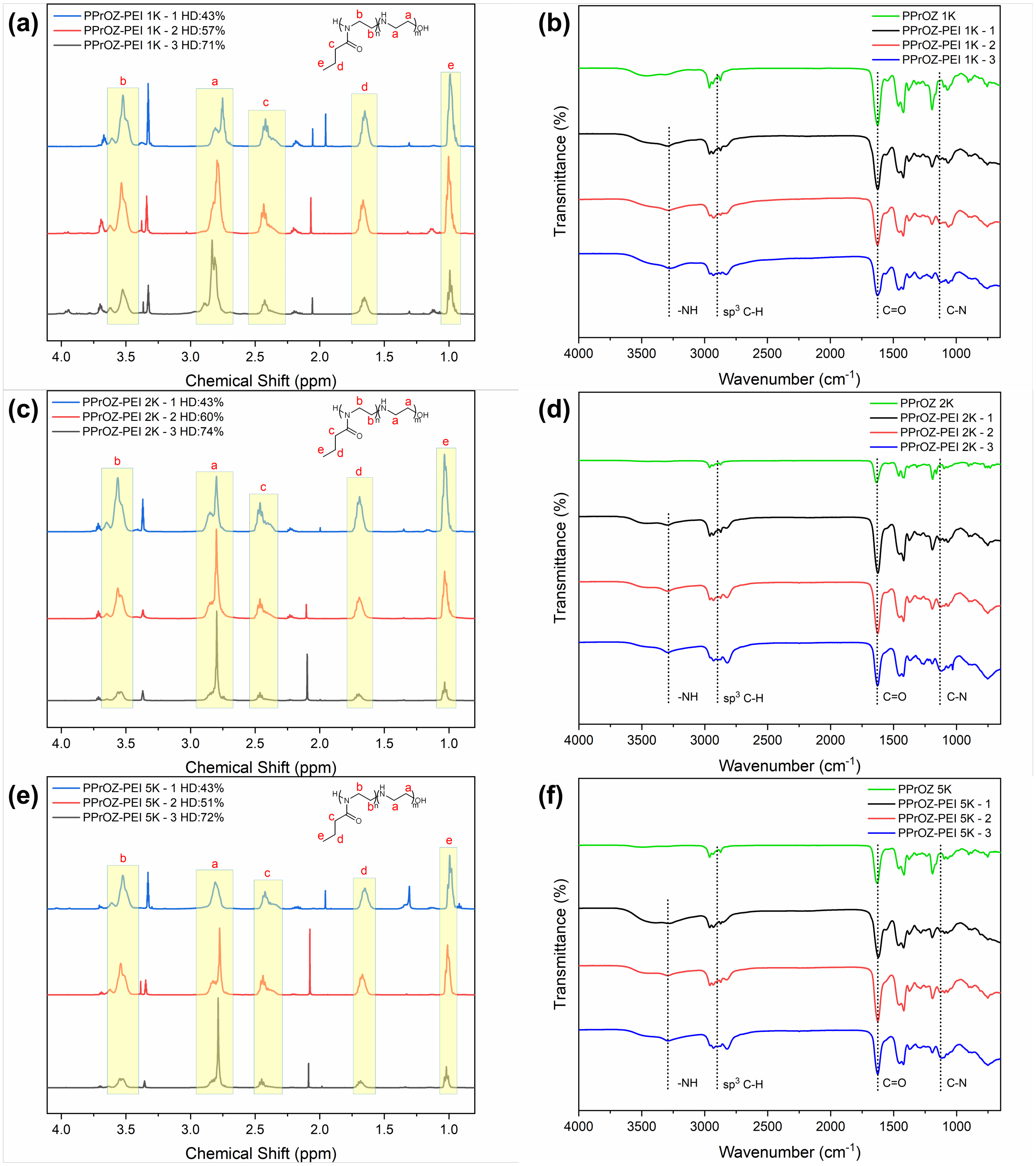

Figure S2. The ^{1}H NMR and FTIR Spectra of (a, b) PPrOZ-PEI 1K, (c,d) PPrOZ-PEI 2K, and (e, f) PPrOZ-PEI 5K Copolymers

2.3. ^{1}H NMR and FTIR Spectra of PPeOZ-PEI 2K and PPeOZ-PEI 5K Copolymers

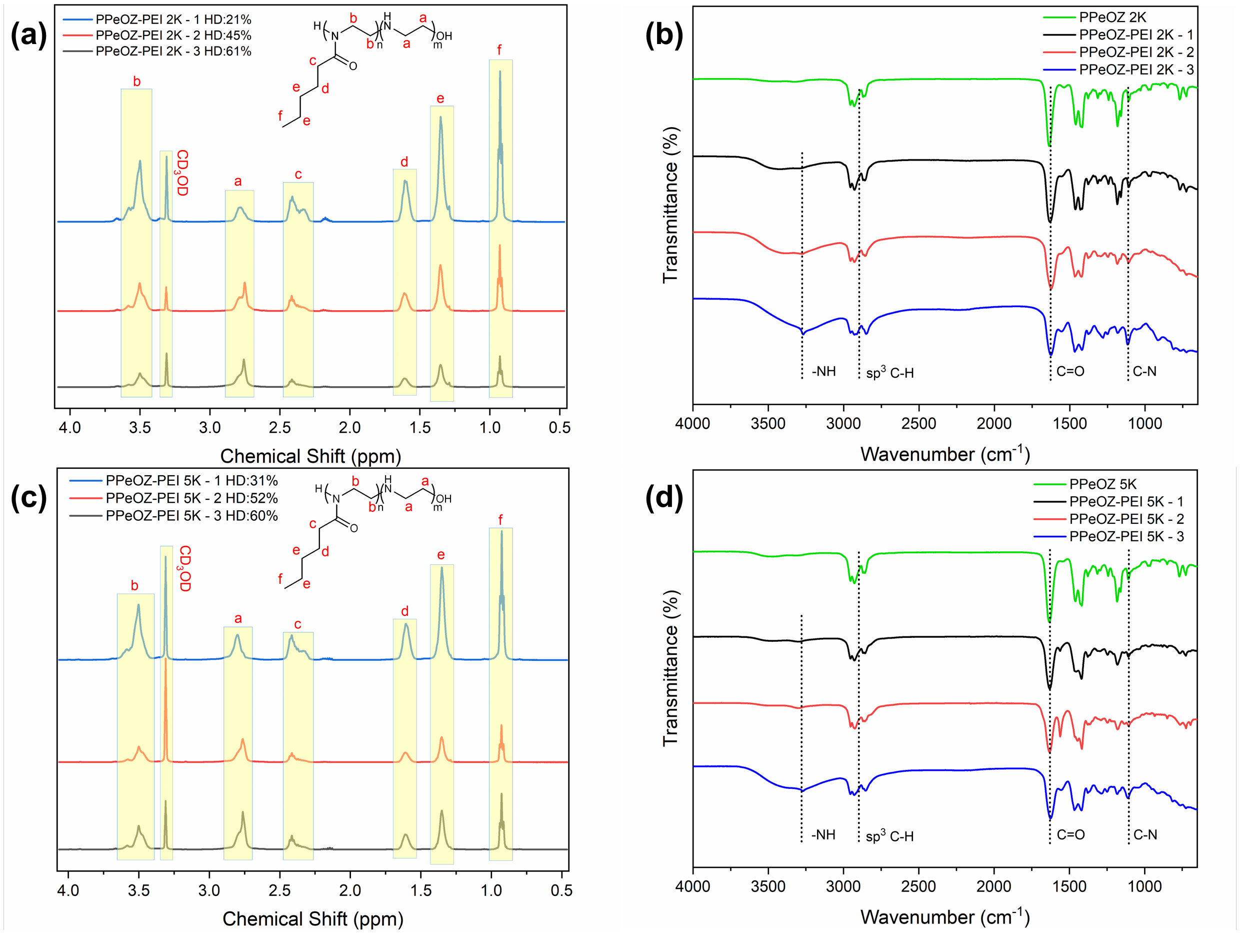

Figure S3. The ^{1}H NMR and FTIR Spectra of (a, b) PPeOZ-PEI 2K and (c,d) PPeOZ-PEI 5K Copolymers

2.4. ^{1}H NMR and FTIR Spectra of PPhOZ-PEI 2K and PPhOZ-PEI 5K Copolymers

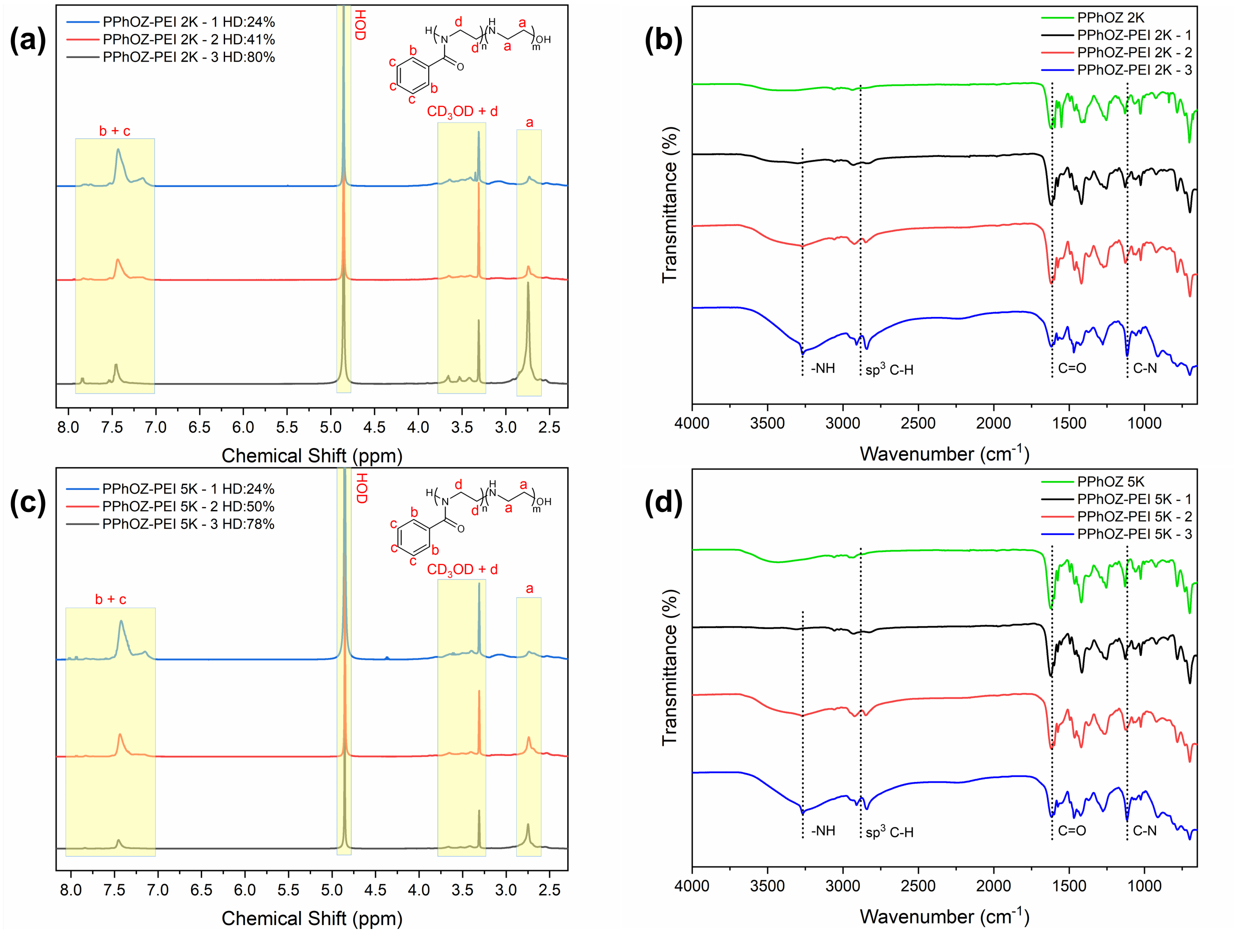

Figure S4. The ^{1}H NMR and FTIR Spectra of (a, b) PPhOZ-PEI 2K and (c,d) PPhOZ-PEI 5K Copolymers

3. DSC Thermograms of PPeOZ-PEI 2K and PPeOZ-PEI 5K Copolymers

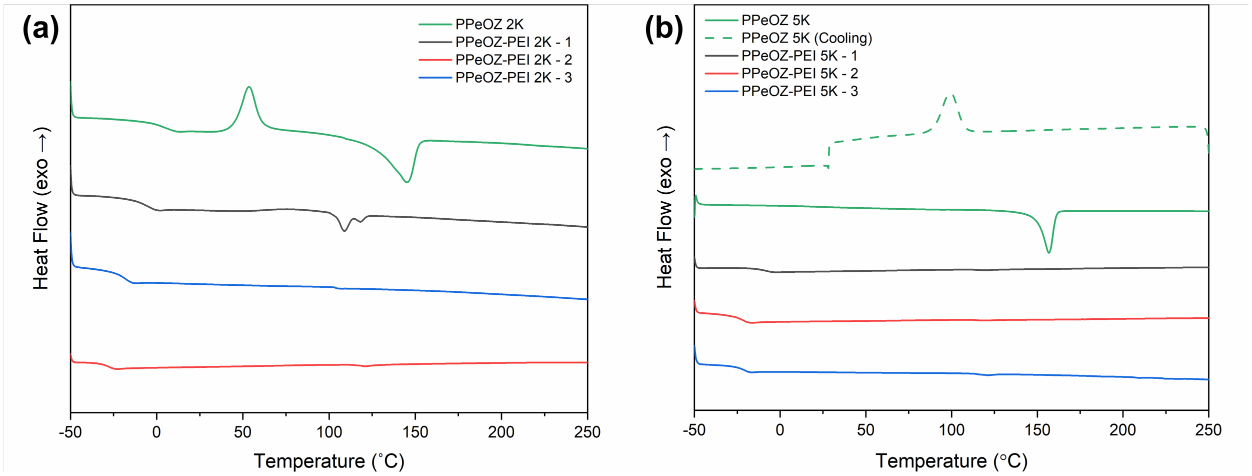

Figure S5. DSC thermograms of (a) PPeOZ-PEI 2K and (b) PPeOZ-PEI 5K at all compositions along with PPeOZ 2K and PPeOZ 5K homopolymers

4. Compositions of Prepared One-Component Epoxy Resins and the Corresponding Amine Hydrogen Equivalent Weight

Table S4. Compositions of Prepared One-Component Epoxy Resins and the Corresponding Amine Hydrogen Equivalent Weight

One-Component Epoxy Resin	Copolymer MW (g/mol)	Number of Secondary Amines	DGEBA (mg)	Copolymer (mg)	AHEW (g/mol)
**PEOZ-PEI 1K - 1 - DGEBA**	825	3	96.8	150	263.4
**PEOZ-PEI 1K - 2 - DGEBA**	706	5	126.5	100	134.4
**PEOZ-PEI 1K - 3 - DGEBA**	593	7	156.4	75	81.5
**PEOZ-PEI 2K - 1 - DGEBA**	1672	6	89.4	150	285.4
**PEOZ-PEI 2K - 2 - DGEBA**	1355	12	144.5	100	117.7
**PEOZ-PEI 2K - 3 - DGEBA**	1231	14	142.3	75	89.6
**PEOZ-PEI 5K - 1 - DGEBA**	4010	18	112.4	150	226.9
**PEOZ-PEI 5K - 2 - DGEBA**	3473	27	133.5	100	127.3
**PEOZ-PEI 5K - 3 - DGEBA**	2766	40	183.9	75	69.3
**PPrOZ-PEI 1K - 1 - DGEBA**	734	4	132.3	150	192.8
**PPrOZ-PEI 1K - 2 - DGEBA**	647	5	132.6	100	128.2
**PPrOZ-PEI 1K - 3 - DGEBA**	560	6	143.0	75	89.2

**PPrOZ-PEI 2K - 1 - DGEBA**	1467	8	132.3	150	192.8
**PPrOZ-PEI 2K - 2 - DGEBA**	1257	11	143.7	100	118.3
**PPrOZ-PEI 2K - 3 - DGEBA**	1083	13	154.2	75	82.7
**PPrOZ-PEI 5K - 1 - DGEBA**	3668	19	132.3	150	192.8
**PPrOZ-PEI 5K - 2 - DGEBA**	3420	23	112.2	100	151.6
**PPrOZ-PEI 5K - 3 - DGEBA**	2770	32	146.6	75	86.9
**PPeOZ-PEI 1K - 1 - DGEBA**	819	2	57.4	150	444.3
**PPeOZ-PEI 1K - 2 - DGEBA**	715	3	69.1	100	245.9
**PPeOZ-PEI 1K - 3 - DGEBA**	479	5	141.7	75	90.0
**PPeOZ-PEI 2K - 1 - DGEBA**	1708	3	44.5	150	573.4
**PPeOZ-PEI 2K - 2 - DGEBA**	1374	6	78.9	100	215.3
**PPeOZ-PEI 2K - 3 - DGEBA**	1152	9	95.8	75	133.1
**PPeOZ-PEI 5K - 1 - DGEBA**	3923	11	71.5	150	356.8
**PPeOZ-PEI 5K - 2 - DGEBA**	3193	18	98.2	100	173.2
**PPeOZ-PEI 5K - 3 - DGEBA**	2915	21	93.1	75	137.0
**PPhOZ-PEI 1K - 1 - DGEBA**	802	2	60.6	150	421.0
**PPhOZ-PEI 1K - 2 - DGEBA**	604	4	107.3	100	158.5
**PPhOZ-PEI 1K - 3 - DGEBA**	469	5	138.6	75	92.0
**PPhOZ-PEI 2K - 1 - DGEBA**	1660	3	50.1	150	508.5
**PPhOZ-PEI 2K - 2 - DGEBA**	1420	6	66.8	100	254.5
**PPhOZ-PEI 2K - 3 - DGEBA**	868	11	159.9	75	79.8
**PPhOZ-PEI 5K - 1 - DGEBA**	4151	8	50.1	150	508.5
**PPhOZ-PEI 5K - 2 - DGEBA**	3231	17	89.5	100	190.0
**PPhOZ-PEI 5K - 3 - DGEBA**	2241	27	151.0	75	84.5

5. DSC Thermograms and Conversion Curves of Prepared One-Component Epoxy Resins

5.1. POZ-PEI 2K One-Component Epoxy Resins

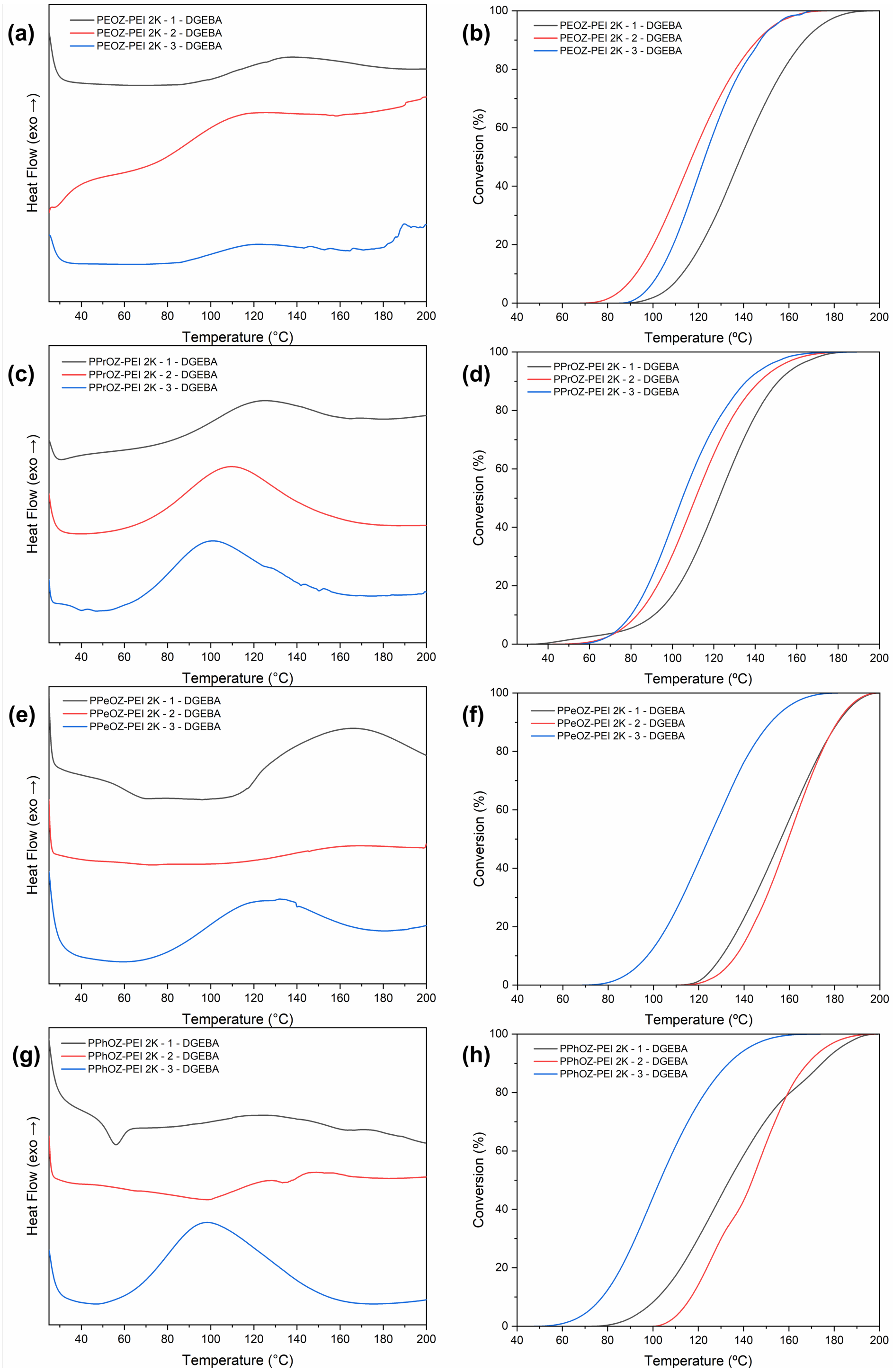

Figure S6. The curing and conversion curves for DGEBA with (a, b) PEOZ-PEI, (c, d) PPrOZ-PEI, (e, f) PPeOZ-PEI, and (g, h) PPhOZ-PEI 1K TLCs at all compositions

5.2. POZ-PEI 5K One-Component Epoxy Resins

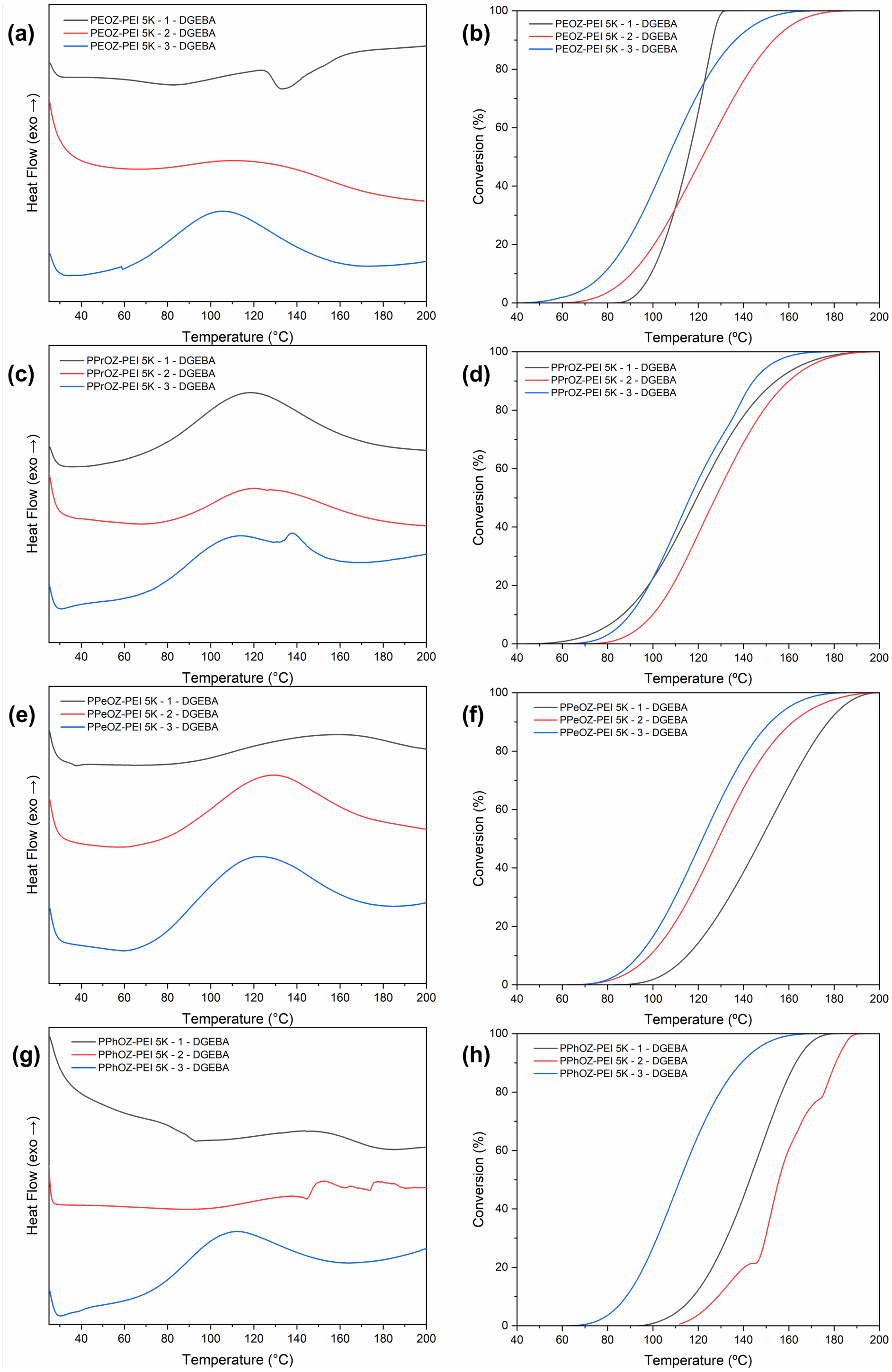

Figure S7. The curing and conversion curves for DGEBA with (a, b) PEOZ-PEI, (c, d) PPrOZ-PEI, (e, f) PPeOZ-PEI, and (g, h) PPhOZ-PEI 5K TLCs at all compositions
